# Core reorganisation in food webs buffers ecosystems against warming

**DOI:** 10.1038/s43247-026-03813-4

**Published:** 2026-07-16

**Authors:** Pablo A. Poleto Antiqueira, Athen Ma, Pavel Kratina, Vito Latora, Lachlan G. Burton, Lei Zhao, Eoin J. O’Gorman

**Affiliations:** 1 School of Life Sciences, University of Essex, Wivenhoe Park, Colchester, UK; 2 Queen Mary University of London, London, UK; 3 Centre for Biodiversity and Sustainability, School of Biological and Behavioural Sciences, Queen Mary University of London, London, UK; 4 Beijing Key Laboratory of Biodiversity and Organic Farming, College of Resources and Environmental Sciences, China Agricultural University, Beijing, China

**Keywords:** Ecological networks, Climate-change ecology, Ecosystem ecology

## Abstract

Food webs are central to ecosystem functioning and stability, yet how they respond to climate warming at the substructural level remains unclear. We used a natural temperature gradient (5–25 °C) spanning 14 whole-stream ecosystems in Iceland to test how warming alters food web substructure organisation (core–periphery) and its consequences for ecosystem functioning (energy flux) and stability (robustness). We found no evidence that warming changed the number or proportion of core and peripheral species, but observed internal reorganisation driven by turnover in core species identity and abundance. While warming consistently decreased robustness to secondary extinctions through reduced food web connectance, core reorganisation enhanced both energy flux and robustness to species loss. Dynamic modelling showed ecosystems were disproportionately sensitive to the loss of core taxa, underscoring their structural importance. These results challenge the view of food webs as fixed hierarchies and reveal an adaptive internal architecture under warming, with core dynamics helping buffer ecosystem responses.

**Figure:**
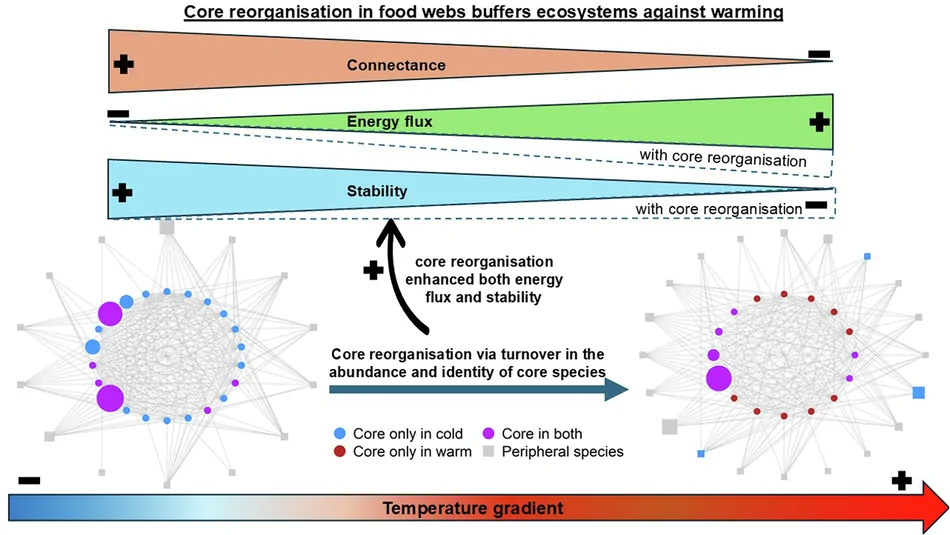

## Abstract (web summary)

Reorganization in food webs under warming reshapes core species, reducing stability through lower connectance but enhancing energy flux and robustness via core dynamics, based on observations across a natural temperature gradient and dynamic modelling.

## Introduction

Ecosystems are structured by complex networks of species interactions that regulate their function, stability, and overall dynamics^1–3^. However, environmental stressors such as global warming are increasingly altering these networks^4–7^, with consequences that are yet to be fully understood because current assessments focus on node-level or simplistic network metrics^8^. Warming affects metabolic rates, phenology, species distributions, trophic interactions, and energy fluxes, potentially destabilising ecological networks^8–11^. As temperatures rise, species at higher trophic levels (predators) typically experience greater metabolic acceleration than those at lower levels, leading to shifts in predator-prey dynamics^12^. Increased metabolic rates in predators can intensify predation pressure^13^, reducing prey populations and disrupting food web structure and stability^6,14^.

Warming also drives changes in species composition through multiple mechanisms. For instance, species with higher thermal tolerance or faster growth rates may outcompete others, leading to biodiversity loss and shifts in community structure^15,16^. Additionally, as thermal conditions become more variable under climate change, generalist species may gain an advantage over specialists, resulting in community homogenisation^17–19^. Finally, warming alters energy flow through ecosystems both directly, by accelerating biochemical reactions^20^, and indirectly, by shifting predator-prey dynamics and altering food web structures^1,8,9^. The overall impact of warming on food webs and ecosystem stability depends on the balance between these direct physiological effects and indirect community-level changes^8^. This balance ultimately determines whether ecosystems remain stable or transition to novel states. Understanding these mechanisms is crucial for predicting future biodiversity patterns and the sustainability of ecosystem services in a warming world.

Although much previous research has focused on the effects that warming has on whole networks^7^, food webs are not homogeneous structures^21,22^. Instead, they exhibit underlying substructures, such as core-periphery organisation, where a subset of highly connected species in the core may play a central role in network stability, relative to less connected species in the periphery^23,24^. The size of the core reflects the network’s structural state: larger cores offer greater potential for link redundancy and rewiring in response to node or interaction loss, while smaller cores suggest increased vulnerability and systems under ecological stress^25–27^. Despite growing recognition of the importance of these core-periphery substructures in ecological networks^23,28,29^, little is known about how warming affects their structural integrity. The core is often composed of functionally important species spanning multiple trophic levels^23^, potentially acting as a stabilising force by maintaining interaction redundancy. However, warming can alter species abundances^30^, interaction strengths^6^, and community composition^31^, which may disproportionately impact the core and its ability to regulate food web dynamics and function. If warming disrupts the core substructure, it could trigger cascading effects throughout the ecosystem, leading to reduced connectivity, altered energy flow, and increased vulnerability to further environmental change^8^.

Here, we use a graph profiling technique^23,32^ to characterise the core-periphery substructure of multiple naturally heated stream ecosystems (see Methods). The streams are located within 1.5 km of each other in the Hengill geothermal valley, Iceland, where they are largely untouched by anthropogenic activity except for occasional sheep grazing. Despite their close proximity and similar physical and chemical characteristics, their temperatures vary from 5 to 25 °C due to geothermal heating of groundwater through the bedrock and remain relatively stable over time^5,33^. Since the streams are located within the same catchment, there are no biogeographical differences typically associated with natural temperature gradients driven by latitude or altitude^5,34^. This system serves as a valuable space-for-time proxy, allowing us to investigate temperature effects on food web substructures in a natural setting while preserving the complexity and realism of natural ecosystems^34^.

We hypothesise (H1) that warming will alter the proportion of species found in the core and periphery of food webs, with the core expected to be more resilient to temperature increases. This is based on the assumption that generalist species—typically found in the core—tend to have a higher thermal tolerance than specialists, which are more commonly located in the periphery. As a result, the relative size of the periphery is expected to decline with warming. Since warming can alter community composition, we predict that (H2) ecological mechanisms such as species replacement and altered abundance will help mitigate the effect of warming on food web substructures. We also expect that (H3) food web stability will decline with increasing temperature, with disproportionately greater losses when core species are lost compared to periphery or random extinctions. Finally, we predict (H4) that warming will alter both ecosystem functioning (total energy flux) and stability (robustness to secondary extinctions), through a combination of direct effects and indirect pathways mediated by changes in network complexity and node dissimilarity.

## Results and discussion

### Core-periphery size

All food webs exhibited a clear core-periphery substructure (Supplementary Figs. 1-2). The core contained species from all trophic levels (Supplementary Table 1) and accounted for an average of 63% of the species, ranging from 33 to 79% depending on the stream. This core size is consistent with other ecological networks (e.g., 50–71% of total network size^23,24^) and significantly larger than those observed in non-ecological networks (5–30% of total network size^32,35^). This suggests that natural ecosystems may exhibit greater linkage redundancy, potentially contributing to their stability.

**Fig. 1:**
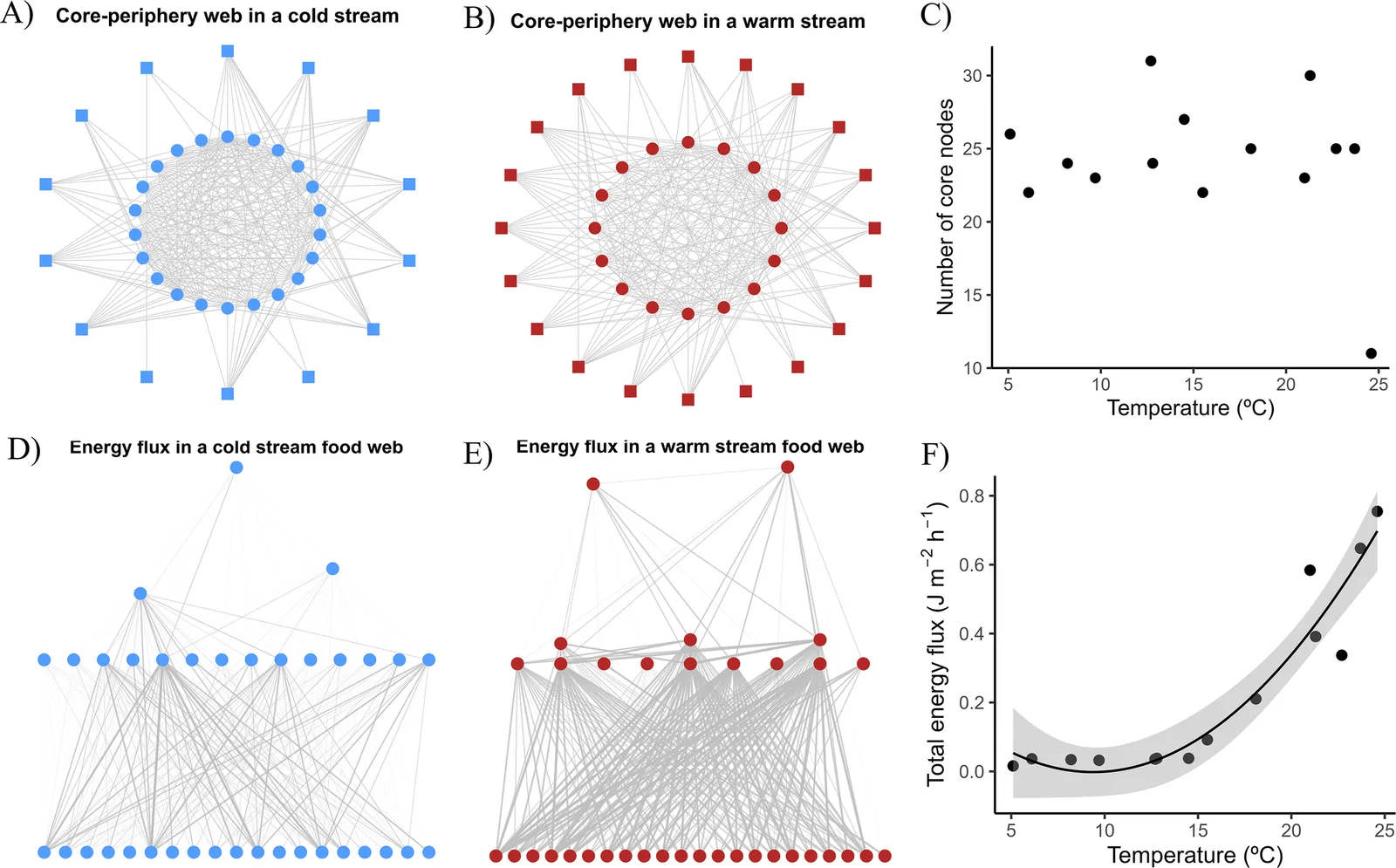
Core–periphery substructure and energy flux through stream food webs along a natural temperature gradient. **A**, **B** Core–periphery substructure of a cold (blue) and warm (red) stream food web, with core nodes (highly connected species) shown as circles in the inner ring and peripheral species as squares in the outer ring. **C** There was no significant relationship between temperature and the number of core nodes across all 14 food webs. **D**, **E** Energy flux within a cold (blue) and warm (red) stream food web, where vertical position reflects trophic level and link thickness indicates the magnitude of energy flux. **F** Total energy flux increased with stream temperature. The solid line represents the fitted relationship from a linear mixed-effects model with a quadratic temperature term (*y* = 0.23 + 0.82*x* + 0.38*x*²), and the shaded area indicates the 95% confidence interval. Temperature had a significant nonlinear effect on total energy flux (Linear mixed models - LME: *F*₂,₁₁ = 55.36, *P* < 0.0001). All food webs are shown in Supplementary Fig. S1.

In contrast to our first hypothesis (H1), we found no statistical evidence that temperature was associated with variation in the number or proportion of core and peripheral species (LME, *P* > 0.05, Fig. 1A–C and Supplementary Fig. 3). This resilience of the core likely stems from the trophic redundancy embedded within ecological networks^23^, which buffers against perturbations^36^ and preserves functional scaffolding^37^ even under elevated temperatures. Our findings align with previous work showing that the core-periphery substructure can remain intact under environmental stressors, even as the taxonomic composition of the core shifts^23,24^. Nevertheless, there was an increase in total energy flux with warming (Fig. 1D–F), indicating an overall increase in the strength of trophic interactions in the warmer environment that could potentially bring destabilising effects^38,39^. This increase was nonlinear (LME, quadratic term: F₂,₁₁ = 55.36, *P* < 0.001), with relatively weak responses at lower temperatures (5–15 °C) and a marked rise at higher temperatures (Fig. 1F), consistent with the temperature dependence of metabolic and interaction rates. We also compared a theory-driven exponential model, following the Metabolic Theory of Ecology^20^, with a quadratic formulation. The exponential model captured the expected monotonic increase in flux with temperature (Supplementary Fig. 4) but provided a poorer fit than the quadratic model (ΔAIC = 22.44). Deviations from strict exponential scaling are consistent with growing evidence that temperature sensitivities (e.g. Q10 temperature coefficient) can vary across environmental gradients rather than remaining constant (e.g.^40–42^). At the ecosystem level, total energy flux represents the rate at which carbon is processed through the food web, linking these estimates to integrative measures of ecosystem functioning. Notably, previous work in the same Hengill stream system has shown that flux-based estimates are strongly correlated with independently derived measures of ecosystem respiration from whole-stream oxygen dynamics^9^, providing empirical support for their ecological realism and relevance as system-level indicators of energy processing.

### Mechanisms underlying warming impacts on core-periphery

**Fig. 2:**
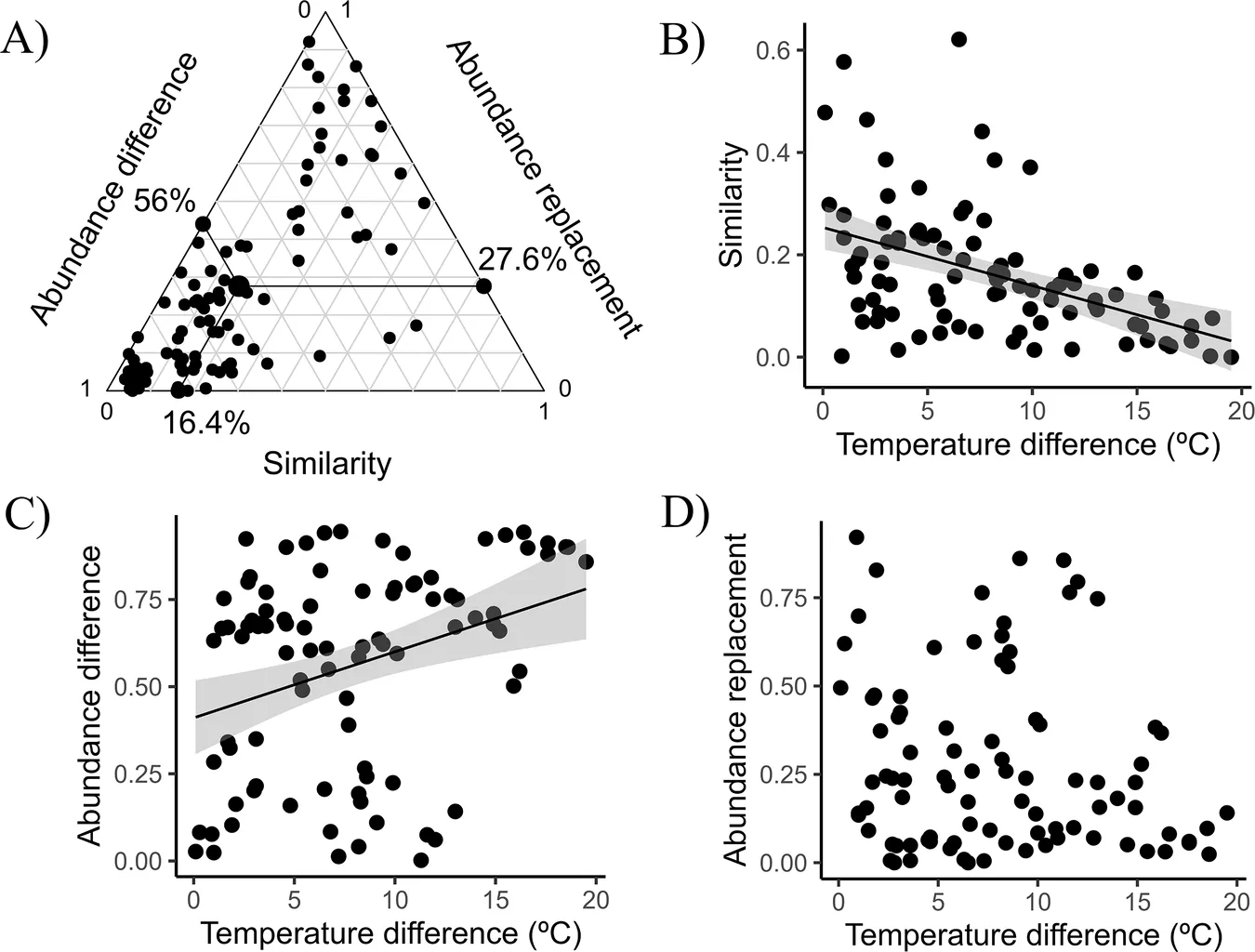
Temperature-driven variation in abundance-based beta diversity of core food web nodes. **A** SDR-simplex plot from pairwise comparisons across the 14 streams, showing the relative contributions of species similarity (1 – total β diversity), abundance replacement, and abundance difference among core nodes. Each point represents a site pair. The large central dot in the triangle represents the centroid of the points. Metrics are based on the Ruzicka dissimilarity matrix for abundance data. Components of beta diversity across the temperature gradient: **B** total similarity (1–total β diversity), **C** abundance difference, and **D** abundance replacement. The solid line represents the fitted relationship from linear models (LM), and the shaded area indicates the 95% confidence interval. Temperature had a significant effect on similarity (*y* = 0.253–0.011*x*; LM: F_1,89_ = 24.05, R^2^ = 0.20, *P* < 0.001) and abundance difference (*y* = 0.410 + 0.019*x*; LM: F_1,89_ = 10.93, R^2^ = 0.10, *P* = 0.001).

We analysed how warming influences species composition and abundance patterns by decomposing total beta diversity (i.e., average node dissimilarity between sites) of the nodes in the complete food web, core, and periphery using species abundance and occurrence metrics^43–45^. In agreement with the second hypothesis (H2), we observed a decline in core similarity with increasing temperature (Fig. 2, Supplementary Fig. 5). When accounting for species abundance, the primary driver of core node dissimilarity was the abundance difference (i.e., the extent to which the total abundance at one site exceeded that of another), which explained 56% of the variation (Fig. 2A). Warming reduced core node similarity (Fig. 2B) primarily by increasing abundance differences among species (Fig. 2C). More specifically, consumer taxa were more abundant in the core as temperature increased, while resource taxa were less abundant, with no change in the periphery (Supplementary Fig. 6). This highlights how a warming-induced strengthening of top-down control, which likely underpinned the observed increase in energy flux (Fig. 1F), was confined to the core food web substructure. There was no detectable impact on abundance replacement (i.e., species abundances at one site being replaced by the abundance of different species at another site; Fig. 2D), indicating that warming does not lead to systematic shifts in the dominant species within the core.

When considering species occurrence instead of abundance, species replacement (i.e., taxonomic turnover) was the dominant driver of dissimilarity in both core nodes and whole food webs, explaining 35% (Supplementary Fig. 5A) and 32% (Supplementary Fig. 8A) of the variation, respectively. Warming reduced similarity in both the core and the entire food web primarily by increasing species replacement, while leaving species richness differences largely unchanged (i.e., differences in the number of species between communities; Supplementary Fig. 5B–D, Supplementary Fig. 8B–D). This indicates that while species identities changed, the overall structure of the food web persisted, largely driven by reorganisation within the core, such that the dominant functional roles were the same but filled by different taxonomic actors^46^. This decoupling of structural persistence and compositional change reveals that ecological network topology can remain ostensibly stable while undergoing deep internal reconfiguration^23,47^. This topological stability reflects a broader ecological principle: that while species identities may change, network architecture often exhibits a form of structural inertia^48,49^, a property tied to emergent system-level organisation and redundancy^50–53^. Such shifts may have far-reaching implications, particularly if replacement species differ in traits, trophic roles, or thermal sensitivity.

### Effects of core-periphery on stability

**Fig. 3:**
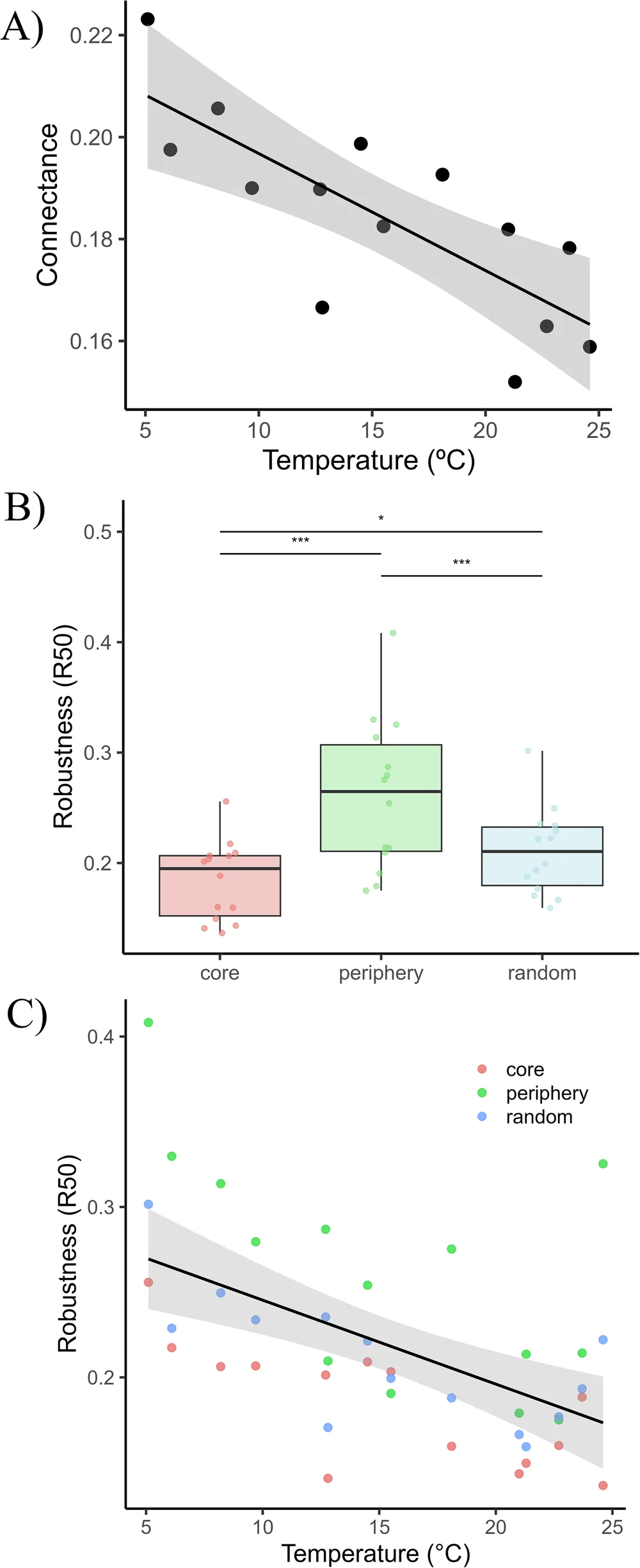
Effects of temperature on food web connectance and robustness. **A** Food web connectance declines with increasing stream temperature (*y* = 0.219–0.002*x*; LME: F_2,26_ = 25.69, *P* < 0.001). **B** Average robustness (*R*_50_) of the 14 food webs under three deletion scenarios: core, periphery, and random. Robustness was significantly lower following core species deletions compared to periphery or random deletions (LME: F_2,26_ = 25.69, *P* < 0.001; Tukey HSD: *p* < 0.05). **C** Robustness decreases with increasing temperature across all deletion scenarios (*y* = 0.260 − 0.005*x₁* + *x₂*; LME: F_1,12_ = 14.74, *P* = 0.002). As no significant interaction was found between temperature and deletion scenario, and an additive model provided a better fit (ΔAIC = 21.64), a single regression line is shown. Shading indicates the 95% confidence interval.

We found that warming reduced food web connectance (Fig. 3A), which is often related to a reduction in robustness to secondary extinctions^54^. Thus, to evaluate the impact of warming on food web stability, we implemented a dynamical model based on energy fluxes to simulate biomass changes in algal producers, detrital taxa, and consumers (see Methods). We applied a dynamic food web model^55^ based on energy inflows and outflows for each taxon, to simulate three species removal scenarios: core first, periphery first, and random. For each sequential deletion, we calculated the robustness *R*_50_, which is the proportion of species subjected to primary removals that resulted in 50% of total species loss^54^. Supporting our third hypothesis (H3), networks were especially vulnerable to the loss of core species, which triggered significantly faster and more extensive secondary extinctions than either periphery-first or random deletions (Fig. 3B). This finding underscores the disproportionate role of core taxa in maintaining network integrity, consistent with expectations from network theory that highlight the core of highly connected nodes in promoting system stability in ecological^23,47^ and non-ecological^26^ networks.

Despite the stabilising role of core inertia, it is important to note that robustness still declined with increasing temperature in all deletion scenarios (Fig. 3C), indicating that warmer streams exhibit lower food web stability regardless of which species are lost. Under a moderate and ecologically realistic warming scenario (5 °C temperature difference), the predicted decline in robustness (0.02–0.03 under random deletions) appears modest in absolute terms. However, relative to the observed range of robustness across streams (0.18–0.27), this represents a 10–15% reduction, indicating a meaningful loss of stability. Moreover, because the studied systems represent long-term, thermally structured communities, responses to rapid climate warming, where species are less likely to be pre-adapted, may lead to stronger declines in both connectance and robustness.

The fact that warming did not alter the proportion of core versus peripheral nodes, but significantly restructured the identity of core species, suggests that ecosystem stability can be eroded even when network topology remains superficially intact. This decoupling between architecture and composition reveals a form of overlooked fragility in ecological networks, where shifting species identities within the network core compromise resilience, even in topologically conserved food webs. Our findings advance the idea that food web stability is governed not only by the presence of a core–periphery substructure, but by the particular species occupying these roles, highlighting the need to monitor functional shifts rather than focusing solely on network topology or species richness.

### Mechanisms underlying warming impacts on functioning and stability

**Fig. 4:**
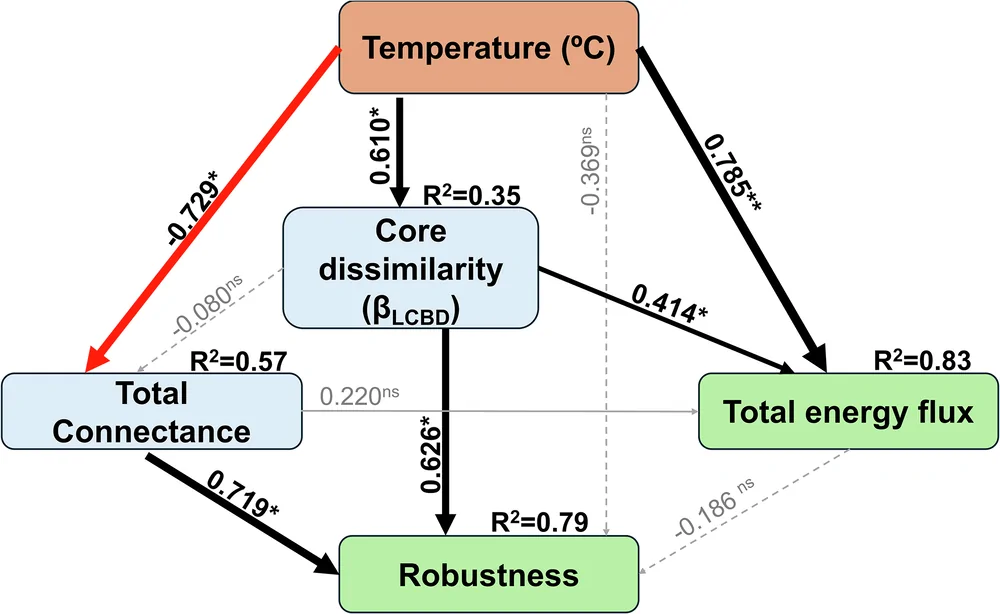
Structural equation model explaining temperature effects on ecosystem functioning (energy flux) and stability (robustness). Standardised path coefficients from piecewise structural equation modelling (pSEM) illustrate the direct and indirect effects of temperature (°C) on core node dissimilarity (β_LCBD_), food web connectance, total energy flux, and robustness. Black and red arrows denote positive and negative relationships, respectively; grey arrows denote non-significant relationships. Arrow thickness corresponds to the magnitude of standardised effects, with asterisks indicating statistical significance (*P < 0.05, **P < 0.01). R^2^ values for component models are given above the boxes of endogenous variables.

We applied structural equation modelling^56^ to evaluate how temperature influences ecosystem functioning (total energy flux) and stability (robustness), both directly and indirectly through its effects on core node identity (core dissimilarity) and food web complexity (connectance). In support of our fourth hypothesis (H4), warming increased total energy flux through both direct and indirect pathways. Warming likely increased energy flux directly by raising the metabolic demands of consumers^20^. The indirect effects of warming on energy flux were mediated by changes in food web substructure. Specifically, warming enhanced core node dissimilarity, which was positively associated with elevated energy flux (Fig. 4; indirect effect = 0.252). This indicates that shifts in the identity and abundance of core taxa may promote greater energy transfer, likely by favouring more metabolically active, interaction-rich species and those with life-history traits adapted to warmer conditions, such as rapid development, multiple generations per year, or flexible reproductive strategies. For example, snails were markedly more abundant in warmer streams, exerting strong top-down control through their efficient grazing of algal biofilms and providing resources for apex fish predators^57,58^. In contrast, these food chains were absent in colder streams, where snails were replaced by smaller-bodied herbivores, and invertebrate omnivores occupied top trophic positions^58^. This shift highlights how warming can restructure trophic hierarchies by favouring thermally tolerant, high-impact taxa that alter energy flow and interaction strength within the ecosystem. Yet, warming exerted only a direct positive effect on energy flux for the complete network and the periphery, with no evidence of mediation via substructural reorganisation (Supplementary Figs. 9–10).

Warming also altered ecosystem stability by reducing food web connectance, which in turn increased robustness to species loss (Fig. 4; indirect effect = –0.524). Concurrently, greater dissimilarity among core species also increased robustness (indirect effect = 0.382), suggesting that turnover in core composition may provide functional insurance against biodiversity loss. However, this stabilising effect was insufficient to offset the destabilising influence of reduced connectance, highlighting a trade-off between structural simplification and taxonomic reorganisation^59^. Notably, such indirect effects of temperature on robustness were absent for both the complete network and the periphery (Supplementary Figs. 9-10), underscoring the unique role of the core in mediating food web responses to warming.

Our bottom-up estimates of energy flux align closely with independent ecosystem-level measurements from these geothermal streams, where warming has been shown to increase gross primary production, consumer biomass, and secondary production across similar temperature gradients^60–62^. This concordance provides empirical support that the temperature-driven increases in energy flux we observe are not artefacts of the modelling framework, but reflect real, system-wide responses. Crucially, however, our approach moves beyond aggregate ecosystem metrics by resolving how energy is redistributed within food webs. We show that warming does not simply increase total flux, but reorganises its distribution across species and trophic interactions, with disproportionate changes occurring within the network core. In doing so, our results bridge the long-standing gap between ecosystem-level flux measurements and network-level structure, revealing a mechanistic pathway through which temperature reshapes both the architecture and functioning of ecological systems.

An important consideration is that the thermal gradient studied here arises from localized geothermal inputs rather than regional climatic differences. As a result, air temperature is consistent across sites, even though many taxa have flying adult stages that may respond to atmospheric conditions. This suggests that the observed differences in community structure and energy flux are primarily driven by variation in aquatic temperature rather than terrestrial climatic effects. In addition, the streams are connected through a shared metacommunity, which facilitates dispersal among sites. Such connectivity may buffer local responses to warming by enabling recolonization and species turnover, in contrast to climate change scenarios where regional warming may simultaneously alter both local environments and the broader species pool. While this limits direct extrapolation to large-scale climate gradients, it provides a powerful framework to isolate the effects of temperature on food-web organisation and ecosystem functioning.

Finally, although our study is based on a single sampling period, previous work in these geothermal streams has shown that temperature-driven patterns in food web structure and functioning are consistent across years, despite seasonal variability^5^. This suggests that the responses we report likely reflect persistent system-level dynamics rather than transient seasonal effects, although incorporating temporal replication would further strengthen this inference.

## Conclusion

Our study reveals how warming drives internal reorganisation of food-web substructures across a broad temperature gradient, particularly within core species that underpin ecosystem functioning and stability. Despite no change in the number and proportion of core and peripheral taxa, warming induced a turnover in core species identity, enhancing energy flux and partially buffering ecosystems against species loss. Critically, the persistence of core size helped offset the destabilising effects of reduced food web connectance, highlighting an underappreciated mechanism of structural resilience. These findings underscore that food webs are not fixed hierarchies but dynamic systems whose internal architecture adapts to environmental change^63^. As such, coarse network metrics like species richness or connectance may mask more nuanced patterns of ecological response. A deeper understanding of food web substructure, particularly the role and composition of core taxa, offers a more powerful framework for predicting ecosystem vulnerability under global change. Prioritising the conservation of functionally pivotal species (e.g., from the core food web) that sustain trophic flows would enhance ecological resilience in an era of accelerated warming. Although derived from geothermal streams, our findings have broad relevance, as core–periphery organisation is a common feature of ecological networks, from terrestrial plant–pollinator systems^47,64^ and agroecosystems^24^ to marine food webs^65^. By linking food web substructure to energy flow and stability, our study bridges community and ecosystem ecology, advancing a general framework for understanding resilience and adaptive capacity in ecological networks. Future research should assess the generality of these patterns across ecosystems of varying complexity and disturbance regimes and examine how multiple global change drivers shape substructural dynamics. For instance, incorporating systems affected by biological invasions represents an important avenue for future research, as invasive species may reorganise core–periphery substructure and thereby alter ecosystem functioning and stability. A deeper understanding of the internal organisation of food webs is therefore critical to anticipating and mitigating the long-term impacts of global change on the biosphere.

## Methods

### Study site

The study was conducted in August 2008 in the Hengill Valley, Iceland (64° 03′ N, 21° 18′ W), a region that has been extensively studied over the past decade^5,6,30,33,66^. We focused on 14 streams located within 1.5 km of each other that are broadly similar in physicochemical properties but span a natural temperature gradient of 5–25 °C^5^. This thermal gradient is due to the indirect heating of groundwater through the bedrock^30,33,57^. The system serves as a space-for-time substitution, enabling the study of eco-evolutionary responses in natural ecosystems under long-term warming conditions, although it does not capture the transient effects that may arise during the warming process^5,30^. Nonetheless, a whole-stream warming experiment conducted within this system demonstrated that changes in community composition along the temperature gradient closely resemble those resulting from the actual warming of a stream^57^.

### Stream sampling

We conducted stream sampling to quantify the three main trophic groups in the ecosystem: benthic diatoms, macroinvertebrates, and fish. Sampling involved collecting diatoms through three stone scrapes per stream, macroinvertebrates via five Surber samples per stream, and fish using three-run depletion electrofishing^5^. Yield-effort curves confirmed our sampling efficiency^5^. Diatoms and macroinvertebrates were identified at the species level under a microscope and counted to estimate population abundance, which was scaled to individuals per m² based on the sampling area. Body mass (mg dry weight) was estimated using linear measurements of at least ten individuals per species and published length-weight relationships^5^. For gut-content analysis, diatoms were identified at the genus level, allowing for the calculation of total abundance and abundance-weighted mean body mass for each genus using species-level data. Throughout the study, all taxa are referred to as species for simplicity. Brown trout (*Salmo trutta*), the only fish species in the system, was weighed on a portable balance, and dry weights were calculated using a wet-to-dry weight conversion^30^. Detailed descriptions of the study system and sampling methods are provided in previous work^5^.

### Food web overview

We conducted extensive gut-content analyses, documenting a total of 49,324 feeding interactions from 1,128 individual consumers collected from the Hengill streams, with the completeness of sampling confirmed by yield-effort curves^5^. We used three distinct methods for describing diets: stomach flushing for fish (5,856 interactions from 109 individuals), acid digestion for herbivorous macroinvertebrates (25,105 interactions from 289 individuals), and gut dissection of macroinvertebrates (18,363 interactions from 730 individuals, including all major functional groups, such as predators, grazers, and detritivores). Fish stomach contents were flushed, immediately preserved in 70% ethanol, and later identified under a microscope. Species-level identification of diatoms in herbivorous macroinvertebrates was achieved by subjecting whole organisms to 62% nitric acid at 65 °C for 18 h to dissolve all organic matter except silicate diatom frustules, enabling precise identification of diatoms, which serve as the primary producers in the streams. A 1 ml subsample of the diatom suspension was pipetted onto a glass coverslip, dried, and fixed onto slides with a drop of Naphrax on a 60 °C hotplate. To identify diatom species, we examined the first 100 diatoms encountered along a continuous, non-overlapping 100-µm-wide transect on the slide, following a fixed route. This approach was sufficient to accurately characterise the diatom species present. Gut dissection was used to quantify predation on other macroinvertebrates as well as feeding interactions involving basal resources other than diatoms. These included coarse particulate organic matter (>1 mm), fine particulate organic matter (<1 mm), macrophytes, filamentous green algae, microscopic green algae, cyanobacteria, and terrestrial subsidies. Invertebrates were dissected under ×20 magnification, and their gut contents were mounted on slides using Hoyer’s medium. Quantification of gut contents was performed by analysing three randomly selected fields of view at ×200 magnification using a compound microscope. Prey items were identified from diagnostic morphological remains (e.g., head capsules, mandibles, setae). While partial digestion can limit taxonomic resolution, this approach reliably distinguishes major prey groups and is widely used in aquatic food web studies.

### Food web construction

To construct a food web for each stream, we began with the species list obtained from the August 2008 sampling. Feeding links were added based on the gut-content analysis of the species present in the studied stream, other streams from the same region, or from literature. Overall, only 12.6% of links were obtained from the literature, while 43.5% were directly observed from the target stream, and the remaining 43.9% were observed from the Hengill region^5^. Among the directly observed links, 74.3% were specific to individual consumer species, with 25.7% inferred from the family level^5^. This hierarchical approach ensured that our food webs achieved an optimal balance of comprehensiveness and precision.

### Food web profiling

The core profiling method identifies a substructure within food webs that consists of species highly interconnected with one another. These species form the core, while less-connected species form the periphery. This method works by ranking species based on the number of their connections (degree) and evaluating the extent to which those connections are linked to more highly connected species^23,32^. Each food web is represented as a binary, undirected network with *S* nodes (species) and *E* links (interactions). To construct the core profile, nodes are ranked in descending order by their degree (i.e., the number of links), *k*_*r*_, where *r* represents the rank of the node. The number of links a node shares with higher-ranked nodes is *k*_*r*_^+^, and the remaining links connecting to lower-ranked nodes are *k*_*r*_ − *k*_*r*_^+^. Starting from the highest-ranked node, *k*_*r*_^+^ fluctuates as nodes further down the rank are included in the analysis, until a point *r*^∗^ is reached where *k*_*r*_^+^ reaches its maximum. Beyond this point, *k*_*r*_^+^ decreases, marking the boundary between the core and the periphery^23^.

### Energy flux estimation

We applied a robust and widely used approach for estimating energy fluxes through trophic links^67^ to predict the effects of increasing temperature on ecosystem-level energy fluxes across different food web profiles (core, periphery, and complete food webs). This method is grounded in the assumption that each stream ecosystem operates at energetic steady state. For all *n* consumer species within a system, this balance can be expressed as:

2 $${G}_{i}={L}_{i},i=1,2,\ldots ,n,$$

where *G*_i_ and *L*_i_ represent the energy gain and loss rates [*J h*^−1^], respectively, for the *i*_th_ species. Basal species are implicitly assumed to maintain energy balance. The two terms in Eq.( 2) can be specified as

3 $${G}_{i}={\sum}_{k\epsilon {R}_{i}}{e}_{{ki}}{w}_{{ki}}{F}_{{ki}}$$

4 $${L}_{i}={Z}_{i}+{\sum}_{j\in {C}_{i}}{w}_{{ij}}{F}_{{ij}}$$

where *R*_*i*_ and *C*_*i*_ are the sets of resource and consumer species, respectively, for the *i*_th_ species. Z_*i*_ represents the population-level energy loss rate due to metabolic activities and mortality [*J h*^−1^]. *F*_*ij*_ denotes the maximum population-level feeding rate of species *j* feeding on species *i* [*J h*^−1^], *e*_*ij*_ is the assimilation efficiency (proportion of energy retained), and *w*_*ij*_ is the consumer’s preference for a specific resource (with the sum of preferences equalling 1 for each consumer). The effective energy flux through a trophic link is therefore given by *e*_*ki*_*w*_*ki*_*F*_*ki*_.

By assuming energy balance (*G*_i_ = *L*_i_) for all species, there are *n* linear equations (corresponding to the *n* consumer species) of the form:

5 $${G}_{i}-{L}_{i}={\sum}_{k\epsilon {R}_{i}}{e}_{{ki}}{w}_{{ki}}{F}_{{ki}}-({Z}_{i}+{\sum}_{j\in {Ci}}{w}_{{ki}}{F}_{{ki}})=0,$$

which can be solved iteratively to obtain the unknown fluxes *F*_*ij;i≠j*_ of all consumer species, provided all the *Z*_*i*_’s, *e*_*ij*_’s, and *w*_*ij*_’s are known.

To implement this, we used the “*fluxing*” function from the *fluxweb* R package, parameterising it with the following data: (1) binary predation matrices for the 14 stream food webs^5^; (2) biomasses for every species in each food web, derived from individual body mass measurements^30^; (3) assimilation efficiencies (*e*_*ij*_’s) adjusted according to established temperature dependencies and resource type (i.e., plant, detritus, or invertebrate)^68^; (4) preferences (*w*_*ij*_’s) depending on resource biomasses; and (5) metabolic rates estimated from the same studies species^9^. Under the energy balance assumption, metabolic losses (Zᵢ) integrate basal, routine, and active components, capturing organismal activity over time. In this study, routine metabolic rates were used as proxies for Zᵢ, which likely underestimates total energy loss. Consequently, flux estimates should be considered conservative (lower bounds). Temperature-dependent parameterisations follow established metabolic theory and are implemented within the widely used fluxweb framework^67^, with prior validation in the same stream food web system^9^. Importantly, these estimates represent the rate of energy transfer between resources and consumers (i.e. interaction strengths), rather than population-level production. As such, they are not directly comparable to metrics such as secondary production or P/B ratios, which describe biomass generation and turnover within taxa. Comparable production-based estimates have been derived independently in the same study system (e.g.^69^).

### Food web dynamics

The food web dynamical model was constructed based on energy budgets that index the carbon fluxes entering and leaving each taxon. The taxa in a food web can be divided into three categories: producers, consumers (including decomposers), and detrital taxa (see^55^ for details). The change in biomass of producers can be described as:

6 $$\frac{d{B}_{i}}{{dt}}={r}_{i}{B}_{i}\left(1-\frac{{\sum }_{j=1}^{{S}_{P}}{c}_{{ij}}{B}_{j}}{K}\right)-{\sum }_{j=h{erbi}}{a}_{{ij}}{w}_{{ij}}{B}_{i}{B}_{j}-{x}_{i}{B}_{i}$$

Here, ‘*herbi*’ are herbivorous taxa, *r* is the maximum specific or intrinsic growth rate, and *S*_*P*_ is the total number of producer taxa. *c*_*ij*_ is the inter-taxon competition coefficient of taxon *j* on taxon *i*, which was randomly drawn from U(0.25, 0.75), consulting a prior study^70^. The carrying capacity *K* was set as the total biomass of all producers multiplied by a factor *f*_*K*_ ~ U(1, 10). *a*_*ij*_ is the attack rate of taxon *j* consuming taxon *i*, which can be derived by (*w*_*ij*_ *F*_*i*j_) / (*B*_*i*_ *B*_*j*_). The metabolic rate *x*_*i*_ was estimated from temperature and size dependencies quantified from the system^9^. For producer taxon *i*, we estimated its total output flux and metabolic rate, deriving the total energy input (i.e. the first term in Eq. 6) and thus estimating growth rate *r*_*i*_ based on energy balance.

The change in biomass of consumers (including herbivores, predators, and decomposers) can be depicted as:

7 $$\frac{d{B}_{i}}{{dt}}={\sum }_{j={res}}{e}_{{ji}}{a}_{{ji}}{w}_{{ji}}{B}_{j}{B}_{i}-{\sum }_{j={pred}}{a}_{{ij}}{w}_{{ij}}{B}_{i}{B}_{j}-{x}_{i}{B}_{i}$$

Here, ‘*res*’ means resource taxon, and ‘*pred*’ means predator taxon. The biomass of predator taxon *i* is increased by assimilation of consumed resources and decreased by predation and respiration. Notice that the change in biomass of decomposers also follows Eq. 7, except that '*res'* means detritus here.

The change in biomass of detrital taxa can be described as:

8 $$\frac{d{B}_{i}}{{dt}}={p}_{i}{\sum }_{j={con}}{\sum }_{k={res}}\left(1-{e}_{{kj}}\right){a}_{{kj}}{B}_{k}{B}_{j}-{\sum }_{j={dec}}{a}_{{ij}}{B}_{i}{B}_{j}$$

Here, ‘*con*’ are consumer taxa and ‘*dec*’ are decomposer taxa, *p*_*i*_ is the proportion of converted detritus *i* in all egestion, and (1 – *e*) is the egestion rate. The biomass stored in detritus *i* is increased by the faeces of consumer taxa, and decreased by consumption of decomposer taxa. Since we estimated the output flux for a detrital taxon *i*, we can calculate the input energy (i.e. the first term in Eq. 8) and thus *p*_*i*_ based on energy balance.

Using the food web dynamical model, we compared three different deletion sequences: (1) randomly removing taxa from the core at each step and only removing taxa from the periphery if there were no more taxa in the core; (2) randomly removing taxa from the periphery at each step and only removing taxa from the core if there were no more taxa in the periphery; and (3) randomly removing taxa from the whole web. We used the 4th order Runge-Kutta method to numerically simulate taxon loss for each food web by sequentially removing taxa. The empirical biomass data were used to give the initial biomass values. A thousand days were simulated after each deletion to record the secondary extinctions. For each sequential deletion, we calculated the robustness *R*_50_, which is the proportion of species subjected to primary removals that resulted in 50% of total species loss^54^. For each food web, 100 replicates were conducted.

### Statistical analysis

We used linear mixed models (LMMs) (“*lme*” function in the “*nlme*” package in R)^71^ to assess how temperature affects multiple response variables, such as the number and proportion of core and periphery species, total energy flux, and connectance. Temperature was included as a fixed explanatory variable, while stream identity was treated as a random effect to account for non-independence among sites. Model assumptions of normality and homoscedasticity were checked through residual visual diagnostics. To evaluate alternative functional forms describing the relationship between temperature and total energy flux, we compared linear, exponential, and quadratic (second-degree polynomial) models. The exponential formulation was implemented by log-transforming energy flux and fitting a linear model against temperature, consistent with predictions from the Metabolic Theory of Ecology^20^. Model performance was assessed using Akaike’s Information Criterion (AIC). The quadratic model provided the best fit (ΔAIC_Polynom-Linear_ = 13, ΔAIC_Polynom-exponential_ = 42.28) and was retained for the main analysis and is presented in the main text (Fig. 1F). The exponential (theory-driven) and linear models are presented in the Supplementary Information as comparative benchmarks (Supplementary Fig. 4).

To investigate how warming affects food web node similarity, we first decomposed total beta diversity (i.e., average node dissimilarity between sites) based on node composition in the complete food web, as well as within core and periphery nodes separately following two approaches: (1) species occurrence (presence/absence data), whereby beta diversity was partitioned into replacement (β_Repl_) and richness difference (β_Richdiff_) components^45^ using the Jaccard dissimilarity coefficient; (2) species abundance, whereby beta diversity was partitioned into abundance replacement (β_Abrepl_) and abundance difference (β_AbDiff_)^44^ using the Ružicka dissimilarity coefficient. We visualised the proportions of each β-diversity component and overall similarity (Sim = 1 – β_total_) using SDR-simplex plots, where each side of the triangular plot represents a different component^44^. The partitioning of beta diversity into its components was performed using the “*beta.div.comp*” function in the “*adespatial*” package^72^, and triangular plots were generated using the “*triangle.plot*” function in the “*ade4*” package^73^. The effects of temperature on each beta diversity component and similarity were analysed using linear models (LMs) (“*lm*” function in “*stats*”).

We used an LMM to assess the effects of temperature and deletion scenario on food web robustness (*R*_50_). The model included temperature, deletion scenario (core-first, periphery-first, and random), and their interaction as fixed effects, with stream identity included as a random effect to account for non-independence among sites. Tukey HSD post hoc tests were conducted to evaluate pairwise differences among deletion scenarios.

Finally, to explore direct and indirect pathways through which temperature influences total energy flux in the food web, we used piecewise structural equation modelling (SEM) (“*psem*” function in the “*piecewiseSEM*” package in R^56^). The model included temperature as an exogenous variable, with core node dissimilarity and total energy flux as endogenous response variables. Additionally, we ran separate models for the complete food web and periphery node dissimilarity to identify structural differences in network responses. Node dissimilarity was estimated using the local contribution to β-diversity (LCBD) method^74^, based on the species abundance dataset, as it is more closely linked to energy flux estimation. Although the direct temperature–flux relationship showed evidence of nonlinearity (ΔAIC > 13, Fig. 1F), both linear and quadratic formulations produced consistent ecological interpretations, characterised by relatively weak responses at lower temperatures and stronger increases at higher temperatures. Therefore, the quadratic model was used to visualise this pattern in Fig. 1F, while the linear formulation was retained in the SEM to ensure comparability among component models, as polynomial terms are not supported within the piecewise SEM framework. This choice does not affect the overall conclusions.

## Supplementary information

Transparent Peer Review file

Supplemental Material

## Data Availability

The data supporting the findings^75^ of this study are available via figshare at https://doi.org/10.6084/m9.figshare.29656244. The deposited dataset contains the main metrics and variables used in the analyses, including the species composition of each food web compartment and the derived energy fluxes. These data are sufficient to reproduce the results reported in the manuscript.

## Code availability

The R code supporting the main findings^75^ of this study is available at https://doi.org/10.6084/m9.figshare.29656244.
