# Supplementary material for: Core reorganisation in food webs buffers ecosystems against warming
Source: Commun Earth Environ. 2026 Jul 16;7(1):874. doi: 10.1038/s43247-026-03813-4 (PMC13745283; doi:10.1038/s43247-026-03813-4)
Supplement: Supplementary file 1 [file 43247_2026_3813_MOESM1_ESM.pdf]

nature portfolio

Peer Review File

Core reorganisation in food webs buffers ecosystems against warming

Corresponding Author: Dr Pablo Augusto Antiqueira

This file contains all reviewer reports in order by version, followed by all author rebuttals in order by version.

Version 0:

Reviewer comments:

Reviewer #1

(Remarks to the Author)
This is a paper addressing the mechanisms whereby climate change may influence biodiversity and food-web dynamics. This is a substantial advance on the normally relatively speculative description of likely effects, as it draws on the strengths of a large data set and tools from graph profiling to quantitatively explore potential changes. The outcomes show the intuitive outcome that disruption of core species particularly impacts stability, and that multiple mechanisms are in operation when warmer conditions impact on food webs. This is an important and novel insight.

The paper is exceptionally well written and provides an elegantly phrased and comprehensive context.

The study system has been well established in previous work.

The figures are highly informative and clearly illustrate the patterns of interest. The analyses are appropriate and well articulated.

While the patterns are clear and make sense there are some difficult-to-resolve issues with the way in which the study is carried out. While the stream temperatures clearly differ, it was not clear to me whether there were any differences in air temperature, noting that many of these taxa have flying adults. In a similar vein, the metacommunity is likely to be shared across sites. This differs from what might be expected under climate change where there would be regional differences in conditions that would act directly on the metacommunity. This is certainly worthy of discussion.

Reviewer #2

(Remarks to the Author)
Comments to Authors
Core reorganisation in food webs buffer ecosystems against warming
Manuscript number COMMSENV-25-6174-T
This manuscript uses data from 14 streams along a natural temperature gradient to inform models testing the effects of temperature on food web assembly, stability, and energy flow. The study streams offer the unique combination of proximity, little landuse disturbance, simple food webs, along with the temperature gradient. The authors presented four hypotheses related to the relationships between temperature and community structure and function. They used multiple modeling methods to quantify temperature effects in the food webs and found changes in the identity and abundance of the core taxa and decreased connectance. This reorganisation of the key taxa in the individual food webs, however, increased energy flow and resilience to localized extinction. They also found that the loss of core taxa from communities reduced system stability.
General comments
I really enjoyed reading and reviewing this manuscript. The work leveraged the uniqueness of the streams in the Hengill Valley, Iceland, and provided insights on relationships between community assemblage, bioenergetics, and stability related to climate change. This work can be used to inform hypotheses about stressor impacts to food webs, in general. It will be interesting to see how these ideas are applied to different systems and stressors.
My only concern about the manuscript is the curious pattern that Figure 1F has, where the slope is approximately zero from

5-15 C. I would recommend checking the model parameters to make sure these temperatures are within the range of acceptable model performance. It might be necessary to change the algorithm.
Detailed comments
L15: It is a little misleading to say that the work tests “how warming alters…” here without mentioning the space-for-time substitution. It IS mentioned in the methods, but I wonder if there is a way to clarify that in the abstract with just a couple words?
L36: I think it is worth noting changing phenology, as that is a universal response to climate change.
L76: These are relatively stable temps, right? Mention for context.
L107: This should be S3a-c, right?
L116: The relationship in Figure1F is really interesting in that the asymptote is y=0 until temp = 15C. What is triggering that increase in the flux at that temperature? Is it relict of the bioenergetics model?
L118-126: Here and throughout, define the regression line and confidence ribbons in your plots. For example, Figure 1F. Also, please provide some information about the significance of relationships.
L201-204: I agree! It bothers me about using richness as a metric of success of restoration projects. Taxa identities matter!
L226: What about favoring taxa with specific life history traits? For example, multi-voltine, parthenogenic, or taxa that can have multiple broods in a year?
L267-270: I appreciate that you mention the role and composition of taxa, as there are still huge gaps in understanding the basic biology of many organisms.
L277-280: Immediately I thought of how this framework would apply to systems with invasive species.
L313: This seems like an abrupt end to the sentence. Maybe add “in previous work5”?
L321: Which functional groups of inverts were dissected?
L323: Please clarify that just a subset of macroinverts were processed using digestion.
L391: Related to my comment on Figure 1, I wonder if the temperature-dependent efficiencies (and metabolic rates) are realistic for your range of temperatures, especially 5-15 C.
Figure S2: For consistency and ease of comparison, it seems like using the same order as the previous figure makes sense.
Figure S4: Are b and d ols regression lines with CI?
Figure S5: It is super interesting that the Eukieefferiella minor was so abundant in the low temperature stream. Any ideas what was going on?
Figure S5: It would help to clarify in the panel that the top plots are core and bottom are periphery. Maybe that is as simple as including “(top)” and “(bottom)” in the bolded title.
Figure S7: It appears that autocorrect changed “Hengill Valley, Iceland,”. Double check all instances.

Reviewer #3

(Remarks to the Author)
This manuscript uses high resolution food webs estimated at one time in a series of temperature-varying (yet other wise similar) streams to show ow food web architecture differs with temperature. It uses a core-satellite approach, a cool idea. The paper also uses modeling experiments to show how changing core and satellite taxa can affect stability, with declining stability with temperature. This paper makes and advance in that it proposes clear structure function links via variation in temperature.

General comments:

1. The sort of food web modeling, especially the dynamic model is bottom up ecosystem ecology. I mean that in a good way and it has a long history dating back to 1970s IBP modelers. Take populations, figure out what they eat, couple with energetics and then sum them up. This paper shows its utility here, great. But it would be nice to see if the upper level fluxes e.g. total animal production, total net and gross primary production are even close to these bottom up estimates. Such studies exist for the same streams, I cited 3 below. Indeed it was odd to me that there has been a decade long investigation of these streams with dozens of papers from another group of researchers , with almost reference to this work (I counted 1 reference) despite that I see that this previous work could have provided a lot of support for the paper that I am reviewing.. Here are some examples that may help provide context for the finding in this paper
https://doi.org/10.1002/ecy.4314
https://doi.org/10.1111/ele.13608
https://doi.org/10.1002/lol2.10244

2. The paper uses language like: "warming reduces..." implying that these streams were warmed up as part of the study. They were not. There are warm streams and cold ones and they have been that way for long time. Thus I would say "warms streams have lower..."

3. Along these lines the streams span a large gradient in temperature (it is why they are so useful for testing such theory). But as predictors of climate change , it is necessary to say how much connectance or robustness will change over a predicted increase in stream temp. Robustness goes from 0.27 to 0.18 over a 20 degree increase in temp. One could say this finding is a good news story because the marginal decline in robustness over say a 5 degree warming would be small (~0.025). Am I right? Is that a small effect?. If the goal of this paper is to provide some idea of climate change effects then I think it should say what these changes mean. A more blunt way of saying this is if temps in streams increase by 20 degrees then we have a lot more to worry about than 50% declines in robustness.

4. Data for this paper came for one time. I understand all too well how much work the gut contents for one time is, so I am not

complaining about he study design. But how might this study look if it were conducted at another time of the growing season?

5. I do not understand the rationale to provide code upon request. That is extra work for authors and requestors alike, when code is so easily archived to e.g., zenodo or even github. I also looked at the data online and are these the raw data for organism abundances and gut contents analyses? It does not appear to be since they seem to be converted to energy? Would it be possible for someone to replicate this study on their compute from the raw data to this paper? If so, then I think the online dataset needs a bit more explanation. If not then raw data should be there to allow a replication.

I looked up Nature's code policy:
"Upon publication, Nature Portfolio journals consider it best practice to release custom computer code in a way that allows readers to repeat the published results. Code should be deposited in a DOI-minting repository such as Zenodo or Code Ocean and cited in the reference list following the guidelines described here. Authors are encouraged to manage subsequent code versions and to use a license approved by the open source initiative. Full details about how the code can be accessed and any restrictions must be described in the Code Availability statement."

Specific comments

17. Cannot say that warming had no effect. For sure it did, but that effect is unquantifiable in the face of the variability.

18 profound, as in a lot?

27. Give this perspective.

77 How does the chemistry differ among the streams? Does it covary with temperature?

107. Cannot say this. See 77. No clear effect of temperature maybe. Null hypothesis test provide no evidence in support of the null. . See the first paragraph in section 2.4 of this paper:
https://doi.org/10.1080/00031305.2018.1527253
Many others say the same thing.

Figure 1 E. This model predicts a negative flux at 5C, which cannot be true, thus this model does not seem like the best one to describe these data. Why not fit an exponential model (following theory) via logging temperature?

Fig 2 b and c Fig 3 a,c. Give the regression parameters and uncertainty here. If the goal is simply to show a linear relationship between x and y and not have a fitted model, then just use correlation.. What is the shade on the plots and how estimated? I am guessing the confidence interval of the model.

195. Warm streams have lower...

211 Despite no evidence for an interaction, I suggest that model with terms (0,1) for periphery and random would fit these data lot better than a single model, thus I suggest plotting 3 lines (unless I am wrong, easy to test).

258. Profound over a giant range of temperature

261. Delete "significant" because it can be confused with $p<0.05$

325 I thought filamentous algae could be dominant in some of these streams?

337 How were prey identified? It is difficult, but certainly possible, to ID prey from parts seen under a microscope

365. 6 years is not very recent.

377, 378. Give units for these. I like to keep rates and fluxes separate for these types of analyses where rate is 1/time and flux is mass/time

398. One way to test these fluxes is to compare the P/B where P is secondary production and B is biomass for these taxa with the empirically measured P/B in the same streams. I provided citations for these data above.

405. The first term in this model should be equivalent to NPP, for which a coarse estimate is 0.5*GPP. Other studies have measured GPP in these same streams. Are they close?

Version 1:

Reviewer comments:

Reviewer #1

(Remarks to the Author)

This is a well considered response to the review and addresses the concerns raised.

Reviewer #2

(Remarks to the Author)
The revised manuscript is much improved over the original submission. The authors addressed my biggest concern from the first iteration. They also made several edits that clarify or add context to the previous version. Additionally, Reviewer 3 made several excellent points in their review which were very insightful and made substantial improvements to the manuscript. From my view, the authors attended to the comments from Reviewer 3, although it is possible that I misinterpreted some details. Throughout this document, my new comments will be preceded by "**"

Reviewer #2 (Remarks to the Author): Comments to Authors Core reorganisation in food webs buffer ecosystems against warming Manuscript number COMMSENV-25-6174-T 3. This manuscript uses data from 14 streams along a natural temperature gradient to inform models testing the effects of temperature on food web assembly, stability, and energy flow. The study streams offer the unique combination of proximity, little landuse disturbance, simple food webs, along with the temperature gradient. The authors presented four hypotheses related to the relationships between temperature and community structure and function. They used multiple modeling methods to quantify temperature effects in the food webs and found changes in the identity and abundance of the core taxa and decreased connectance. This reorganisation of the key taxa in the individual food webs, however, increased energy flow and resilience to localized extinction. They also found that the loss of core taxa from communities reduced system stability. R3: Thank you for the thorough and constructive evaluation of our manuscript, as well as the positive assessment of its novelty and contribution. We are particularly grateful for the insightful comments and suggestions, which have helped us improve both the clarity and robustness of the manuscript. Below, we provide a detailed, point-by-point response.

General comments 4. I really enjoyed reading and reviewing this manuscript. The work leveraged the uniqueness of the streams in the Hengill Valley, Iceland, and provided insights on relationships between community assemblage, bioenergetics, and stability related to climate change. This work can be used to inform hypotheses about stressor impacts to food webs, in general. It will be interesting to see how these ideas are applied to different systems and stressors. My only concern about the manuscript is the curious pattern that Figure 1F has, where the slope is approximately zero from 5-15 C. I would recommend checking the model parameters to make sure these temperatures are within the range of acceptable model performance. It might be necessary to change the algorithm. R4: To address your concern regarding the shape of the relationship in Figure 1F, we re-evaluated the model specification by comparing alternative functional forms. A quadratic polynomial model (lme(eflux_complete ~ poly(temp, 2), random = ~1 | ID)) provided a better fit than the linear model (lme(eflux_complete ~ temp, random = ~1 | ID)), as indicated by a lower AIC (–10.07 vs. 3.43, ΔAIC > 13), supporting a degree of nonlinearity in the relationship between temperature and total energy flux. However, both models yielded qualitatively similar patterns and led to consistent ecological interpretations.
Importantly, the apparent plateau at lower temperatures (5–15 °C) reflects a relatively weak temperature dependence within this range, followed by a stronger response at higher temperatures, rather than a model artefact. The polynomial model captures this pattern more flexibly but does not alter the overall conclusions.
Based on this comparison, we have updated Figure 1F to present the fitted relationship from the polynomial model, which better represents the observed pattern. However, we retained the linear formulation in the piecewise SEM analyses to ensure model consistency and comparability across component models, as polynomial terms are not supported within this framework. We now clarify this distinction in the Results/Discussion (L120-123) and Methods (L520-524 and L553-561) sections.
Revised text – Results/Discussion:
"This increase was nonlinear (LME, quadratic term: $F_{2,11} = 55.36$, $P < 0.001$), with relatively weak responses at lower temperatures (5–15 °C) and a marked rise at higher temperatures (Fig. 1f), consistent with the temperature dependence of metabolic and interaction rates."
Revised text - Methods:
"To evaluate the form of the relationship between temperature and total energy flux, we compared linear and quadratic (second-degree polynomial) formulations. The polynomial model provided a better fit (ΔAIC > 13, Fig. 1F), indicating some nonlinearity, although both approaches yielded qualitatively similar patterns."
…
"Although the direct temperature–flux relationship showed evidence of nonlinearity (ΔAIC > 13, Fig. 1F), both linear and quadratic formulations produced consistent ecological interpretations, characterised by relatively weak responses at lower temperatures and stronger increases at higher temperatures. Therefore, the quadratic model was used to visualise this pattern in Fig. 1F, while the linear formulation was retained in the SEM to ensure comparability among component models, as polynomial terms are not supported within the piecewise SEM framework. This choice does not affect the overall conclusions."

** I appreciate the follow-through related to my concerns about the model fit as plotted in Figure 1F. The updated model reflects the non-linear relationship between temperature and biological processes. I would expect that the function would be exponential, with the flux=0 at temp=0, instead of a second order polynomial based on Q10, but there is evidence that Q10 does not follow a strict exponential relationship (see Mundim et al. 2020, https://doi.org/10.1016/j.ecolmodel.2020.109127, for more information. That said, the polynomial relationship you modelled is likely indistinguishable from an exponential relationship at the lower values, given the error represented in your plot. This model represents a much better fit than the original, linear relationship you modelled so it addresses my concerns.

Detailed comments 5. L15: It is a little misleading to say that the work tests "how warming alters…" here without mentioning

the space-for-time substitution. It IS mentioned in the methods, but I wonder if there is a way to clarify that in the abstract with just a couple words? R5: We have revised the abstract to explicitly state that the temperature gradient arises from geothermal heating of groundwater and represents a space-for-time proxy for long-term warming (L19-L20).
Revised text:
"This gradient, generated by indirect geothermal heating of groundwater, provides a space-for-time proxy for long-term warming."

**Thank you for the clarification.

6. L36: I think it is worth noting changing phenology, as that is a universal response to climate change.
R6: We have revised the sentence accordingly to explicitly include changes in phenology alongside other well-established ecological responses to warming (L38).

**Thank you for the clarification.

7. L76: These are relatively stable temps, right? Mention for context. R7: We have revised the text to mention that temperatures are relatively stable over time due to the geothermal origin of the gradient (L78-80).
Revised text:
"Despite their close proximity and similar physical and chemical characteristics, their temperatures vary from 5 to 25 °C due to geothermal heating of groundwater through the bedrock and remain relatively stable over time".

**Thank you for adding that context.

8. L107: This should be S3a-c, right? R8: We have now corrected the citation accordingly (now Fig. S3a-c, line 112).

**Thank you for making that correction.

9. L116: The relationship in Figure1F is really interesting in that the asymptote is y=0 until temp = 15C. What is triggering that increase in the flux at that temperature? Is it relict of the bioenergetics model? R9: Thank you for highlighting this interesting pattern. We replied to this point in more detail in response to your main commentary above. We noted that the apparent plateau at lower temperatures (5–15 °C) is not a strict asymptote at zero but reflects a weak temperature dependence of energy flux within this range. The increase in flux at higher temperatures emerges from the combined effects of temperature-dependent metabolic and consumption rates in the bioenergetic framework, which scale nonlinearly with temperature.
Importantly, this pattern is not an artefact of the modelling approach. Re-fitting the relationship using alternative functional forms (linear vs. quadratic) yielded qualitatively consistent results, with the polynomial model providing a better fit (ΔAIC > 13) while preserving the same overall interpretation. We have clarified this in the revised manuscript (see the Response 4).

**See my response above. Thank you for the additional information here.

10. L118-126: Here and throughout, define the regression line and confidence ribbons in your plots. For example, Figure 1F. Also, please provide some information about the significance of relationships. R10: We have revised the figure legends to explicitly define the regression lines and confidence intervals shown in the plots. Specifically, we now clarify that solid lines represent fitted relationships from linear mixed-effects models and shaded areas indicate 95% confidence intervals. In addition, we now report the statistical significance of the relationships. For Figure 1F, we used a quadratic formulation to better capture the nonlinear pattern
observed in the data ($y= 0.23 + 0.82x + 0.38x^2$), which provided a significantly improved fit compared to a linear model (ΔAIC > 13; LMM: $F_{2,11} = 55.36$, $P < 0.0001$).

**Thank you for clarifying those captions.

11. L201-204: I agree! It bothers me about using richness as a metric of success of restoration projects. Taxa identities matter! R11: Thank you for the positive feedback.

12. L226: What about favoring taxa with specific life history traits? For example, multi-voltine, parthenogenic, or taxa that can have multiple broods in a year? R12: We agree that life-history traits are likely an important mechanism underlying the observed patterns. We have revised the text to acknowledge that warming may favour taxa with traits such as rapid reproduction and multiple generations per year, which can contribute to increased energy flux (L248-252).
Revised text:
"This indicates that shifts in the identity and abundance of core taxa may promote greater energy transfer, likely by favouring more metabolically active, interaction-rich species and those with life-history traits adapted to warmer conditions, such as rapid development, multiple generations per year, or flexible reproductive strategies."

**Thank you for clarifying.

13. L267-270: I appreciate that you mention the role and composition of taxa, as there are still huge gaps in understanding the basic biology of many organisms. R13: Thank you, we agree that significant knowledge gaps remain regarding the basic biology of many taxa, particularly in complex food webs.

14. L277-280: Immediately I thought of how this framework would apply to systems with invasive species. R14: We agree that systems with invasive species provide a particularly relevant context to test this framework. In response, we have explicitly included biological invasions as an important dimension of global change for future studies (L338-L345), noting that invasive species may reorganise core–periphery substructure and alter ecosystem functioning and stability. We believe this addition strengthens the manuscript by highlighting a promising avenue for future research.
Revised text:
"Future research should assess the generality of these patterns across ecosystems of varying complexity and disturbance regimes and examine how multiple global change drivers shape substructural dynamics. For instance, incorporating systems affected by biological invasions represents an important avenue for future research, as invasive species may reorganise core–periphery substructure and thereby alter ecosystem functioning and stability. A deeper understanding of the internal organisation of food webs is therefore critical to anticipating and mitigating the long-term impacts of global change on the biosphere."

**Excellent. Including that idea broadens the potential reach of your work.

15. L313: This seems like an abrupt end to the sentence. Maybe add "in previous work[5]"? R15: We added the text as suggested (L376).

**Thank you for the clarification.

16. L321: Which functional groups of inverts were dissected? R16: We now note that these dissections included all major functional groups (L383-385). Revised text: "18,363 interactions from 730 individuals, including all major functional groups, such as predators, grazers, and detritivores"

**Thank you for the clarification.

17. L323: Please clarify that just a subset of macroinverts were processed using digestion. R17: We now clarify that this process was only used for species-level identification of diatoms in herbivorous macroinvertebrates (L386-390). Revised text: "Species-level identification of diatoms in herbivorous macroinvertebrates was achieved by subjecting whole organisms to 62% nitric acid at 65 °C for 18 hours to dissolve all organic matter except silicate diatom frustules, enabling precise identification of diatoms, which serve as the primary producers in the streams."

**Thank you for including that additional information about the methods.

18. L391: Related to my comment on Figure 1, I wonder if the temperature-dependent efficiencies (and metabolic rates) are realistic for your range of temperatures, especially 5-15 C. R18: We agree that assessing the realism of temperature-dependent efficiencies and metabolic rates across the observed thermal range is important for interpreting the patterns in Fig. 1. The temperature-dependent parameterisations used in our energy flux calculations are based on established metabolic theory and implemented in the widely used fluxweb framework (Gauzens et al. 2019), and have been previously validated in the same stream food web system (Kordas et al. 2022). Importantly, metabolic rates were estimated using the same or closely related taxa sampled along the same in situ temperature gradient, which increases the ecological realism and reliability of the parameterisation for this system. We added this information in the Methods (L467-470)
Gauzens, B., Barnes, A., Giling, D.P., Hines, J., Jochum, M., Lefcheck, J.S., Rosenbaum, B., Wang, S. and Brose, U., 2019. fluxweb: An R package to easily estimate energy fluxes in food webs. Methods in Ecology and Evolution, 10(2), pp.270-279.
Kordas, R.L., Pawar, S., Kontopoulos, D.G., Woodward, G. and O'Gorman, E.J., 2022. Metabolic plasticity can amplify ecosystem responses to global warming. Nature Communications, 13(1), p.2161.

**Thank you for providing this context to the methods.

19. Figure S2: For consistency and ease of comparison, it seems like using the same order as the previous figure makes sense. R19: We altered the order as suggested.

**Thank you for making that change.

20. Figure S4: Are b and d ols regression lines with CI? R20: The solid line represents the fitted relationship from linear models (LM), and the shaded area indicates the 95% confidence interval. Temperature had a significant effect on similarity ($y = 0.613 - 0.015x$; LM: $F_{1,89} = 41.67$, $R^2 = 0.31$, $P < 0.001$) and species replacement ($y = 0.247 + 0.013x$; LM: $F_{1,89} = 22.47$, $R^2 = 0.19$, $P < 0.001$). We added this information in the figure S4 legend.

**This is super helpful for the reader. Thanks for adding.

21. Figure S5: It is super interesting that the Eukieefferiella minor was so abundant in the low temperature stream. Any ideas what was going on? R21: We do not know for sure, but we suspect this is due to a lack of competition from larger grazers such as snails, which are particularly efficient at rasping biofilms off rocks as their abundance increases in warmer streams. We have added a clarification to the Figure S5 legend.
Revised text: "The chironomid Eukiefferiella minor was super-abundant in the coldest stream (possibly due to lack of competition from larger diatom grazers such as the snail Radix balthica)"

**Very interesting. Thank you for the additional information!

22. Figure S5: It would help to clarify in the panel that the top plots are core and bottom are periphery. Maybe that is as simple as including “(top)” and “(bottom)” in the bolded title. R22: We have revised Figure S5 to clarify the panel structure by indicating “(top)” and “(bottom)” in the legend bolded title as suggested, making it explicit that the upper panels correspond to core components and the lower panels to periphery components

**Thank you for including that clarification.

23. Figure S7: It appears that autocorrect changed “Hengill Valley, Iceland,”. Double check all instances. R23: We have double-checked the terminology and confirm that it is correct. However, we have revised the legends of Figs. S4 and S7 and removed the mentioned text to improve consistency with similar figures in the manuscript (e.g., Fig. 2 and Fig. S6).

**Sorry for the error in my previous review.

Reviewer #3

(Remarks to the Author)
This manuscript is a revision of one that I reviewed before. The authors addressed my most substantial criticisms. I also checked out the code and data and indeed it was easy to download and peruse, thanks for that.

I also read through my review where I found an awful mistake in my advice:

"Figure 1 E. This model predicts a negative flux at 5C, which cannot be true, thus this model does not seem like the best one to describe these data. Why not fit an exponential model (following theory) via logging temperature?"

I meant logging energy flux (i.e. exponential model)! Logging temperature is a terrible idea, apologies for any confusion. But I would still fit an exponential model as that follows better from theory. Brown et al. 2004, Ecology. Even if it does have \Delta AIC higher than that from a quadratic. I view the goal of this paper is is to test theory, not compare statistical models. Deviations from theory (via a less than perfect fit) are where we learn.

This answer needs better justification "While direct validation using empirical P/B ratios would be informative, such data were not consistently available for all taxa. The fluxweb approach provides a mechanistically grounded and widely validated framework that integrates metabolic theory and trophic structure."

I agree with the authors that such data are not available for all taxa. But they exist for common taxa and indeed for the entire assemblage (Table 1 Nelson et al. Ecology). How does productivity and thus P/B derived from the model compare for some of the common taxa or most of the assemblage? I am not looking for a close relationship. But if off by 100 or 1000, then I would want to know why.

Ditto on the NPP. I am not sure what "conceptually linked to NPP" means. The sum producer production should be something less than NPP (given not all photoautotrophs were assessed), and NPP is probably close to 0.5* GPP. Again, the goal is to not for these fluxes to closely match heree, but not off by 1000 either.

Open Access This Peer Review File is licensed under a Creative Commons Attribution 4.0 International License, which permits use, sharing, adaptation, distribution and reproduction in any medium or format, as long as you give appropriate credit to the original author(s) and the source, provide a link to the Creative Commons license, and indicate if changes were made.
In cases where reviewers are anonymous, credit should be given to 'Anonymous Referee' and the source.
The images or other third party material in this Peer Review File are included in the article's Creative Commons license, unless indicated otherwise in a credit line to the material. If material is not included in the article's Creative Commons license and your intended use is not permitted by statutory regulation or exceeds the permitted use, you will need to obtain permission directly from the copyright holder.
To view a copy of this license, visit https://creativecommons.org/licenses/by/4.0/

Response Letter – Antiqueira et al. Core reorganisation in food webs buffers ecosystems against warming - Communications Earth & Environment – R1

Editor comments

Dear Dr Antiqueira,

Your manuscript titled "Core reorganisation in food webs buffers ecosystems against warming" has now been seen by 3 reviewers, and we include their comments at the end of this message. They find your work of interest, but some important points are raised. We are interested in the possibility of publishing your study in Communications Earth & Environment, but would like to consider your responses to these concerns and assess a revised manuscript before we make a final decision on publication. In revision, please include a full discussion of the results in the context of previous work, as suggested by reviewer #3.

We therefore invite you to revise and resubmit your manuscript, along with a point-by-point response that takes into account the points raised. Please highlight all changes in the manuscript text file.

Please submit your point-by-point responses as a separate file, distinct from your cover letter where you can add responses to the Editors' comments that you do not want to be made available to the reviewers. Word files are preferred. We recommend that any figures, tables or graphs that are included in the response to reviewers are also included in the main article or Supplementary Information.

We are committed to providing a fair and constructive peer-review process. Please don't hesitate to contact us if you wish to discuss the revision in more detail.

R: Dear Communications Earth & Environment Editor,

Thank you for the opportunity to revise and resubmit our manuscript entitled "*Core reorganisation in food webs buffers ecosystems against warming*". We appreciate the thoughtful and constructive comments provided by you and the reviewers, which have helped us to improve the quality and clarity of the manuscript.

We have now revised the manuscript accordingly and addressed all comments in detail in a point-by-point response document. All changes in the manuscript have been clearly highlighted, as requested.

We hope that the revised version meets the journal's expectations and we look forward to your assessment. Thank you again for your consideration.

Author's Note: Below, we provide detailed replies to each point raised by the reviewers. Our responses are presented in boldface, and we indicate the line numbers corresponding to the clean version of the revised manuscript where the changes were made. All modifications within the clean version are highlighted in boldface to enhance clarity and traceability.

REVIEWER COMMENTS:

Reviewer #1 (Remarks to the Author):

1. This is a paper addressing the mechanisms whereby climate change may influence biodiversity and food-web dynamics. This is a substantial advance on the normally relatively speculative description of likely effects, as it draws on the strengths of a large data set and tools from graph profiling to quantitatively explore potential changes. The outcomes show the intuitive outcome that disruption of core species particularly impacts stability, and that multiple mechanisms are in operation when warmer conditions impact on food webs. This is an important and novel insight.
The paper is exceptionally well written and provides an elegantly phrased and comprehensive context.
The study system has been well established in previous work.
The figures are highly informative and clearly illustrate the patterns of interest. The analyses are appropriate and well articulated.

R1: Thank you for the careful reading of our manuscript and for the positive and encouraging evaluation of our work. We appreciate the recognition of the novelty of our approach, the robustness of the dataset, and the clarity of the presentation. Below, we address the specific comment raised.

2. While the patterns are clear and make sense there are some difficult-to-resolve issues with the way in which the study is carried out. While the stream temperatures clearly differ, it was not clear to me whether there were any differences in air temperature, noting that many of these taxa have flying adults. In a similar vein, the metacommunity is likely to be shared across sites. This differs from what might be expected under climate change where there would be regional differences in conditions that would act directly on the metacommunity. This is certainly worthy of discussion.

R2: All study streams are located within a relatively small geographic area and therefore experience highly similar air temperature regimes. In contrast, the observed thermal gradient is driven by localized geothermal inputs, which generate substantial variation in stream and soil temperature while minimizing differences in atmospheric conditions among sites. Although many taxa have flying adult stages and may be influenced by air temperature, the limited variation in

atmospheric conditions suggests that the observed patterns primarily reflect differences in aquatic thermal environments.

We agree that the shared metacommunity across streams represents an important distinction from many climate change scenarios, where regional warming may simultaneously affect both local environments and the broader species pool. In our system, dispersal among sites likely maintains connectivity and may buffer local biodiversity responses through recolonization and species turnover. At the same time, this configuration allows us to isolate the effects of temperature on food-web structure and functioning while holding the regional species pool relatively constant.

We have now added a new paragraph in the Discussion to explicitly acknowledge these points and to clarify both the strengths and limitations of our space-for-time substitution approach in the context of climate change (L297-309).

Revised text:

"An important consideration is that the thermal gradient studied here arises from localized geothermal inputs rather than regional climatic differences. As a result, air temperature is consistent across sites, even though many taxa have flying adult stages that may respond to atmospheric conditions. This suggests that the observed differences in community structure and energy flux are primarily driven by variation in aquatic temperature rather than terrestrial climatic effects. In addition, the streams are connected through a shared metacommunity, which facilitates dispersal among sites. Such connectivity may buffer local responses to warming by enabling recolonization and species turnover, in contrast to climate change scenarios where regional warming may simultaneously alter both local environments and the broader species pool. While this limits direct extrapolation to large-scale climate gradients, it provides a powerful framework to isolate the effects of temperature on food-web organisation and ecosystem functioning."

Reviewer #2 (Remarks to the Author):

Comments to Authors
Core reorganisation in food webs buffer ecosystems against warming
Manuscript number COMMSENV-25-6174-T
3. This manuscript uses data from 14 streams along a natural temperature gradient to inform models testing the effects of temperature on food web assembly, stability, and energy flow. The study streams offer the unique combination of proximity, little landuse disturbance, simple food webs, along with the temperature gradient. The authors presented four hypotheses related to the relationships between temperature and community structure and function. They used multiple modeling methods to quantify temperature effects in the food webs and found changes in the identity and abundance of the core taxa and decreased connectance. This reorganisation of the key taxa in the

individual food webs, however, increased energy flow and resilience to localized extinction. They also found that the loss of core taxa from communities reduced system stability.

R3: Thank you for the thorough and constructive evaluation of our manuscript, as well as the positive assessment of its novelty and contribution. We are particularly grateful for the insightful comments and suggestions, which have helped us improve both the clarity and robustness of the manuscript. Below, we provide a detailed, point-by-point response.

General comments

4. I really enjoyed reading and reviewing this manuscript. The work leveraged the uniqueness of the streams in the Hengill Valley, Iceland, and provided insights on relationships between community assemblage, bioenergetics, and stability related to climate change. This work can be used to inform hypotheses about stressor impacts to food webs, in general. It will be interesting to see how these ideas are applied to different systems and stressors.

My only concern about the manuscript is the curious pattern that Figure 1F has, where the slope is approximately zero from 5-15 C. I would recommend checking the model parameters to make sure these temperatures are within the range of acceptable model performance. It might be necessary to change the algorithm.

R4: To address your concern regarding the shape of the relationship in Figure 1F, we re-evaluated the model specification by comparing alternative functional forms. A quadratic polynomial model (*lme(eflux_complete ~ poly(temp, 2), random = ~1 | ID)*) provided a better fit than the linear model (*lme(eflux_complete ~ temp, random = ~1 | ID)*), as indicated by a lower AIC (−10.07 vs. 3.43, ΔAIC > 13), supporting a degree of nonlinearity in the relationship between temperature and total energy flux. However, both models yielded qualitatively similar patterns and led to consistent ecological interpretations.

Importantly, the apparent plateau at lower temperatures (5–15 °C) reflects a relatively weak temperature dependence within this range, followed by a stronger response at higher temperatures, rather than a model artefact. The polynomial model captures this pattern more flexibly but does not alter the overall conclusions.

Based on this comparison, we have updated Figure 1F to present the fitted relationship from the polynomial model, which better represents the observed pattern. However, we retained the linear formulation in the piecewise SEM analyses to ensure model consistency and comparability across component models, as polynomial terms are not supported within this framework. We now clarify this distinction in the Results/Discussion (L120-123) and Methods (L520-524 and L553-561) sections.

Revised text – Results/Discussion:

“This increase was nonlinear (LME, quadratic term: $F_{2,11}$ = 55.36, P < 0.001), with relatively weak responses at lower temperatures (5–15 °C) and a marked rise at higher temperatures (Fig. 1f), consistent with the temperature dependence of metabolic and interaction rates.”

Revised text - Methods:

“To evaluate the form of the relationship between temperature and total energy flux, we compared linear and quadratic (second-degree polynomial) formulations. The polynomial model provided a better fit (ΔAIC > 13, Fig. 1F), indicating some nonlinearity, although both approaches yielded qualitatively similar patterns.”

…

“Although the direct temperature–flux relationship showed evidence of nonlinearity (ΔAIC > 13, Fig. 1F), both linear and quadratic formulations produced consistent ecological interpretations, characterised by relatively weak responses at lower temperatures and stronger increases at higher temperatures. Therefore, the quadratic model was used to visualise this pattern in Fig. 1F, while the linear formulation was retained in the SEM to ensure comparability among component models, as polynomial terms are not supported within the piecewise SEM framework. This choice does not affect the overall conclusions.”

Detailed comments

5. L15: It is a little misleading to say that the work tests “how warming alters…” here without mentioning the space-for-time substitution. It IS mentioned in the methods, but I wonder if there is a way to clarify that in the abstract with just a couple words?

R5: We have revised the abstract to explicitly state that the temperature gradient arises from geothermal heating of groundwater and represents a space-for-time proxy for long-term warming (L19-L20).

Revised text:

“This gradient, generated by indirect geothermal heating of groundwater, provides a space-for-time proxy for long-term warming.”

6. L36: I think it is worth noting changing phenology, as that is a universal response to climate change.

R6: We have revised the sentence accordingly to explicitly include changes in phenology alongside other well-established ecological responses to warming (L38).

7. L76: These are relatively stable temps, right? Mention for context.
R7: We have revised the text to mention that temperatures are relatively stable over time due to the geothermal origin of the gradient (L78-80).

Revised text:

"Despite their close proximity and similar physical and chemical characteristics, their temperatures vary from 5 to 25 °C due to geothermal heating of groundwater through the bedrock and remain relatively stable over time".

8. L107: This should be S3a-c, right?

R8: We have now corrected the citation accordingly (now Fig. S3a-c, line 112).

9. L116: The relationship in Figure1F is really interesting in that the asymptote is y=0 until temp = 15C. What is triggering that increase in the flux at that temperature? Is it relict of the bioenergetics model?

R9: T**hank you for highlighting this interesting pattern. We replied to this point in more detail in response to your main commentary above. We noted that the apparent plateau at lower temperatures (5–15 °C) is not a strict asymptote at zero but reflects a weak temperature dependence of energy flux within this range. The increase in flux at higher temperatures emerges from the combined effects of temperature-dependent metabolic and consumption rates in the bioenergetic framework, which scale nonlinearly with temperature.**

Importantly, this pattern is not an artefact of the modelling approach. Re-fitting the relationship using alternative functional forms (linear vs. quadratic) yielded qualitatively consistent results, with the polynomial model providing a better fit (ΔAIC > 13) while preserving the same overall interpretation. We have clarified this in the revised manuscript (see the Response 4).

10. L118-126: Here and throughout, define the regression line and confidence ribbons in your plots. For example, Figure 1F. Also, please provide some information about the significance of relationships.

R10: We have revised the figure legends to explicitly define the regression lines and confidence intervals shown in the plots. Specifically, we now clarify that solid lines represent fitted relationships from linear mixed-effects models and shaded areas indicate 95% confidence intervals.

In addition, we now report the statistical significance of the relationships. For Figure 1F, we used a quadratic formulation to better capture the nonlinear pattern

observed in the data (y= 0.23 + 0.82x + 0.38x^2), which provided a significantly improved fit compared to a linear model (ΔAIC > 13; LMM: $F_{2,11}$ = 55.36, P < 0.0001).

11. L201-204: I agree! It bothers me about using richness as a metric of success of restoration projects. Taxa identities matter!

R11: Thank you for the positive feedback.

12. L226: What about favoring taxa with specific life history traits? For example, multi-voltine, parthenogenic, or taxa that can have multiple broods in a year?

R12: We agree that life-history traits are likely an important mechanism underlying the observed patterns. We have revised the text to acknowledge that warming may favour taxa with traits such as rapid reproduction and multiple generations per year, which can contribute to increased energy flux (L248-252).

Revised text:

"This indicates that shifts in the identity and abundance of core taxa may promote greater energy transfer, likely by favouring more metabolically active, interaction-rich species and those with life-history traits adapted to warmer conditions, such as rapid development, multiple generations per year, or flexible reproductive strategies."

13. L267-270: I appreciate that you mention the role and composition of taxa, as there are still huge gaps in understanding the basic biology of many organisms.

R13: Thank you, we agree that significant knowledge gaps remain regarding the basic biology of many taxa, particularly in complex food webs.

14. L277-280: Immediately I thought of how this framework would apply to systems with invasive species.

R14: We agree that systems with invasive species provide a particularly relevant context to test this framework. In response, we have explicitly included biological invasions as an important dimension of global change for future studies (L338-L345), noting that invasive species may reorganise core–periphery substructure and alter ecosystem functioning and stability. We believe this addition strengthens the manuscript by highlighting a promising avenue for future research.

Revised text:

“Future research should assess the generality of these patterns across ecosystems of varying complexity and disturbance regimes and examine how multiple global change drivers shape substructural dynamics. For instance, incorporating systems affected by biological invasions represents an important avenue for future research, as invasive species may reorganise core–periphery substructure and thereby alter ecosystem functioning and stability. A deeper understanding of the internal organisation of food webs is therefore critical to anticipating and mitigating the long-term impacts of global change on the biosphere.”

15. L313: This seems like an abrupt end to the sentence. Maybe add “in previous work5”?

R15: We added the text as suggested (L376).

16. L321: Which functional groups of inverts were dissected?

R16: We now note that these dissections included all major functional groups (L383-385).

Revised text:

“18,363 interactions from 730 individuals, including all major functional groups, such as predators, grazers, and detritivores”

17. L323: Please clarify that just a subset of macroinverts were processed using digestion.

R17: We now clarify that this process was only used for species-level identification of diatoms in herbivorous macroinvertebrates (L386-390).

Revised text:

“Species-level identification of diatoms in herbivorous macroinvertebrates was achieved by subjecting whole organisms to 62% nitric acid at 65 °C for 18 hours to dissolve all organic matter except silicate diatom frustules, enabling precise identification of diatoms, which serve as the primary producers in the streams.”

18. L391: Related to my comment on Figure 1, I wonder if the temperature-dependent efficiencies (and metabolic rates) are realistic for your range of temperatures, especially 5-15 C.

R18: We agree that assessing the realism of temperature-dependent efficiencies and metabolic rates across the observed thermal range is important for

interpreting the patterns in Fig. 1. The temperature-dependent parameterisations used in our energy flux calculations are based on established metabolic theory and implemented in the widely used fluxweb framework (Gauzens et al. 2019), and have been previously validated in the same stream food web system (Kordas et al. 2022). Importantly, metabolic rates were estimated using the same or closely related taxa sampled along the same in situ temperature gradient, which increases the ecological realism and reliability of the parameterisation for this system. We added this information in the Methods (L467-470)

Gauzens, B., Barnes, A., Giling, D.P., Hines, J., Jochum, M., Lefcheck, J.S., Rosenbaum, B., Wang, S. and Brose, U., 2019. fluxweb: An R package to easily estimate energy fluxes in food webs. Methods in Ecology and Evolution, 10(2), pp.270-279.

Kordas, R.L., Pawar, S., Kontopoulos, D.G., Woodward, G. and O'Gorman, E.J., 2022. Metabolic plasticity can amplify ecosystem responses to global warming. Nature Communications, 13(1), p.2161.

19. Figure S2: For consistency and ease of comparison, it seems like using the same order as the previous figure makes sense.

R19: We altered the order as suggested.

20. Figure S4: Are b and d ols regression lines with CI?

R20: The solid line represents the fitted relationship from linear models (LM), and the shaded area indicates the 95% confidence interval. Temperature had a significant effect on similarity (y = 0.613 - 0.015x; LM: $F_{1,89}$ = 41.67, R^2 = 0.31, $P < 0.001$) and species replacement (y = 0.247 + 0.013x; LM: $F_{1,89}$ = 22.47, R^2 = 0.19, $P < 0.001$). We added this information in the figure S4 legend.

21. Figure S5: It is super interesting that the Eukieefferiella minor was so abundant in the low temperature stream. Any ideas what was going on?

R21: We do not know for sure, but we suspect this is due to a lack of competition from larger grazers such as snails, which are particularly efficient at rasping biofilms off rocks as their abundance increases in warmer streams. We have added a clarification to the Figure S5 legend.

Revised text:

"The chironomid *Eukiefferiella minor* was super-abundant in the coldest stream (possibly due to lack of competition from larger diatom grazers such as the snail *Radix balthica*)"

22. Figure S5: It would help to clarify in the panel that the top plots are core and bottom are periphery. Maybe that is as simple as including “(top)” and “(bottom)” in the bolded title.

R22: We have revised Figure S5 to clarify the panel structure by indicating “(top)” and “(bottom)” in the legend bolded title as suggested, making it explicit that the upper panels correspond to core components and the lower panels to periphery components

23. Figure S7: It appears that autocorrect changed “Hengill Valley, Iceland,”. Double check all instances.

R23: We have double-checked the terminology and confirm that it is correct. However, we have revised the legends of Figs. S4 and S7 and removed the mentioned text to improve consistency with similar figures in the manuscript (e.g., Fig. 2 and Fig. S6).

Reviewer #3 (Remarks to the Author):

24. This manuscript uses high resolution food webs estimated at one time in a series of temperature-varying (yet other wise similar) streams to show ow food web architecture differs with temperature. It uses a core-satellite approach, a cool idea. The paper also uses modeling experiments to show how changing core and satellite taxa can affect stability, with declining stability with temperature. This paper makes and advance in that it proposes clear structure function links via variation in temperature.

R24: Thank you for the careful, detailed, and thoughtful evaluation of our manuscript. We particularly appreciate the recognition that our study advances structure–function links in food webs along temperature gradients. You raise several important points that have helped us substantially improve the manuscript, especially in terms of contextualization, interpretation, and transparency. Below, we address each comment in detail.

General comments:
25. 1. The sort of food web modeling, especially the dynamic model is bottom up ecosystem ecology. I mean that in a good way and it has a long history dating back to 1970s IBP modelers. Take populations, figure out what they eat, couple with energetics and then sum them up. This paper shows its utility here, great. But it would be nice to see if the upper level fluxes e.g. total animal production, total net and gross primary

production are even close to these bottom up estimates. Such studies exist for the same streams, I cited 3 below. Indeed it was odd to me that there has been a decade long investigation of these streams with dozens of papers from another group of researchers , with almost reference to this work (I counted 1 reference) despite that I see that this previous work could have provided a lot of support for the paper that I am reviewing.. Here are some examples that may help provide context for the finding in this paper
https://doi.org/10.1002/ecy.4314
https://doi.org/10.1111/ele.13608
https://doi.org/10.1002/lol2.10244

R25: Thank you for this insightful comment and for highlighting the importance of previous ecosystem-level work in these streams. We have now explicitly incorporated the suggested studies into a new paragraph in the Discussion (L283-296), clarifying how our bottom-up estimates compare with independently measured ecosystem processes. These studies report consistent increases in primary production and consumer biomass with temperature, supporting the magnitude and direction of our flux estimates. We further emphasise that our contribution lies in resolving how energy is redistributed within food webs under warming, thereby linking ecosystem-level fluxes with network-level organisation.

Revised text:

"Our bottom-up estimates of energy flux align closely with independent ecosystem-level measurements from these geothermal streams, where warming has been shown to increase gross primary production, consumer biomass, and secondary production across similar temperature gradients[58–60]. This concordance provides empirical support that the temperature-driven increases in energy flux we observe are not artefacts of the modelling framework, but reflect real, system-wide responses. Crucially, however, our approach moves beyond aggregate ecosystem metrics by resolving how energy is redistributed within food webs. We show that warming does not simply increase total flux, but reorganises its distribution across species and trophic interactions, with disproportionate changes occurring within the network core. In doing so, our results bridge the long-standing gap between ecosystem-level flux measurements and network-level structure, revealing a mechanistic pathway through which temperature reshapes both the architecture and functioning of ecological systems."

26. 2. The paper uses language like: "warming reduces..." implying that these streams were warmed up as part of the study. They were not. There are warm streams and cold ones and they have been that way for long time. Thus I would say "warms streams have lower..."

R26: We have revised the manuscript to clarify interpretation, improve statistical rigor, and refine the presentation of our results. Specifically, we have revised the manuscript throughout to avoid causal language implying experimental

manipulation. We now consistently refer to variation along a natural temperature gradient. For instance, we replaced "warming reduces…" with "warmer streams exhibit…"

27. 3. Along these lines the streams span a large gradient in temperature (it is why they are so useful for testing such theory). But as predictors of climate change , it is necessary to say how much connectance or robustness will change over a predicted increase in stream temp. Robustness goes from 0.27 to 0.18 over a 20 degree increase in temp. One could say this finding is a good news story because the marginal decline in robustness over say a 5 degree warming would be small (~0.025). Am I right? Is that a small effect?. If the goal of this paper is to provide some idea of climate change effects then I think it should say what these changes mean. A more blunt way of saying this is if temps in streams increase by 20 degrees then we have a lot more to worry about than 50% declines in robustness.

R27: We have revised the manuscript to explicitly report robustness changes and their ecological implications. In our geothermal stream systems (composed by long-term warming-adapted communities) robustness (R50) declined from ~0.27 to ~0.18 across the 20°C gradient (for the random deletion scenario). Considering a moderate warming (~5°C intervals), the predicted reduction in robustness (~0.025) may appear small in raw value, but it represents a 10–15% decline relative to observed stream robustness, indicating a substantial reduction in network stability even in these resilient systems.

In typical climate change scenarios, where most species are not pre-adapted to warmer conditions, warming could have much stronger effects on both connectance and robustness. Importantly, warming responses were compartment-specific: core species and their links were consistently the most vulnerable, whereas periphery species and overall network structure were comparatively more resistant. These points have been incorporated into the revised Results/Discussion section (L207-L217) to clarify the magnitude and ecological significance of warming effects across different compartments of the food web.

Revised text:

"Despite the stabilising role of core inertia, it is important to note that robustness still declined with increasing temperature in all deletion scenarios (Fig. 3c), indicating that warmer streams exhibit lower food web stability regardless of which species are lost. Under a moderate and ecologically realistic warming scenario (5 °C temperature difference), the predicted decline in robustness (0.02–0.03 under random deletions) appears modest in absolute terms. However, relative to the observed range of robustness across streams (0.18–0.27), this represents a 10–15% reduction, indicating a meaningful loss of stability. Moreover, because the studied systems represent long-term, thermally structured communities, responses to rapid climate warming, where species are less likely to be pre-adapted, may lead

to stronger declines in both connectance and robustness.”

28. 4. Data for this paper came for one time. I understand all too well how much work the gut contents for one time is, so I am not complaining about he study design. But how might this study look if it were conducted at another time of the growing season?

R28: We acknowledge that our data represent a single snapshot in time, and that seasonal variation could influence food web structure and energy fluxes. While resolving temporal dynamics at this level of detail remains challenging, previous work in these geothermal streams has shown that key food web responses to temperature are consistent across sampling periods. In particular, O'Gorman et al. 2019 found that temperature-driven patterns in food web structure and functioning were robust across years, despite seasonal variability. These findings suggest that the temperature effects we report likely capture persistent, system-level responses rather than transient seasonal patterns. Nevertheless, we agree that incorporating temporal replication would be a valuable direction for future research, particularly to assess how seasonal dynamics interact with warming to shape food web reorganisation. These points have been incorporated into the revised Results/Discussion section (L310-L315)

O’Gorman, E. J., et al. "A simple model predicts how warming simplifies wild food webs." ***Nature Climate Change*** **9.8 (2019): 611-616. https://doi.org/10.1038/s41558-019-0513-x**

Revised text:

“Finally, although our study is based on a single sampling period, previous work in these geothermal streams has shown that temperature-driven patterns in food web structure and functioning are consistent across years, despite seasonal variability5. This suggests that the responses we report likely reflect persistent system-level dynamics rather than transient seasonal effects, although incorporating temporal replication would further strengthen this inference.”

29. 5. I do not understand the rationale to provide code upon request. That is extra work for authors and requestors alike, when code is so easily archived to e.g., zenodo or even github. I also looked at the data online and are these the raw data for organism abundances and gut contents analyses? It does not appear to be since they seem to be converted to energy? Would it be possible for someone to replicate this study on their compute from the raw data to this paper? If so, then I think the online dataset needs a bit more explanation. If not then raw data should be there to allow a replication.

I looked up Nature's code policy:
"Upon publication, Nature Portfolio journals consider it best practice to release custom

computer code in a way that allows readers to repeat the published results. Code should be deposited in a DOI-minting repository such as Zenodo or Code Ocean and cited in the reference list following the guidelines described here. Authors are encouraged to manage subsequent code versions and to use a license approved by the open source initiative. Full details about how the code can be accessed and any restrictions must be described in the Code Availability statement."

R29: We agree that transparent data and code availability are essential for reproducibility. In line with this, we have now deposited the R scripts used for the main analyses and figures in a public repository (link: https://doi.org/10.6084/m9.figshare.29656244) and updated the Code Availability statement accordingly (L563-572).

The dataset provided includes all key variables and derived metrics used in the analyses, including food web structure, energy fluxes, and species composition within each food web compartment. We confirm that these data are sufficient to fully reproduce the analyses, figures and results presented in the manuscript.

The raw data on organism abundances and gut contents were used exclusively to construct the food webs and derive the energy flux estimates. These underlying datasets follow established protocols that have been extensively applied in previous studies of these systems (e.g., O'Gorman et al. 2019; Kordas et al. 2022), and thus are not unique to this study. We have clarified this distinction in the revised Data Availability statement and expanded the dataset description to improve transparency.

We believe that, with the inclusion of the analysis code and the processed data, the study is fully reproducible. Nevertheless, we are happy to provide additional details on the raw data and processing steps if required.

Specific comments

30. 17. Cannot say that warming had no effect. For sure it did, but that effect is unquantifiable in the face of the variability.

R30: We agree that failure to detect a statistical effect does not imply absence of an effect. We have revised the wording to reflect lack of evidence rather than absence of effect (L20-23).

Revised text:

"We found no evidence that warming was associated with changes in the number or proportion of core and peripheral species, but observed internal reorganization driven by turnover in core species identity and abundance."

31. 18 profound, as in a lot?

R31: We improve the sentence as follows (L22-23):

"…but observed internal reorganization driven by turnover in core species identity and abundance."

32. 27. Give this perspective.

R32: We have clarified the ecological context and explicitly framed the contribution relative to existing work (L28-31).

Revised text:

"These findings challenge the traditional view of food webs as fixed hierarchies, highlighting instead their adaptive internal architecture under environmental change. By identifying core reorganisation as a key mechanism, our results provide a new perspective on how ecosystems may buffer climate impacts."

33. 77 How does the chemistry differ among the streams? Does it covary with temperature?

R33: Previous work conducted in the same catchment and time period has shown no systematic variation in key physicochemical variables along the temperature gradient, indicating that temperature is largely decoupled from other environmental drivers in this system (O'Gorman et al. 2019). We added a text to make this point clear (L350-352).

Added text (Methods):

"We focused on 14 streams located within 1.5 km of each other that are broadly similar in physicochemical properties but span a natural temperature gradient of 5–25 °C."

O'Gorman, E.J., Petchey, O.L., Faulkner, K.J., Gallo, B., Gordon, T.A., Neto-Cerejeira, J., Ólafsson, J.S., Pichler, D.E., Thompson, M.S. and Woodward, G., 2019. A simple model predicts how warming simplifies wild food webs. Nature Climate Change, 9(8), pp.611-616.

34. 107. Cannot say this. See 77. No clear effect of temperature maybe. Null hypothesis test provide no evidence in support of the null. . See the first paragraph in section 2.4 of this paper:
https://doi.org/10.1080/00031305.2018.1527253
Many others say the same thing.

R34: We agree and have revised the language to avoid accepting the null hypothesis (L110-112).

Revised text:

"In contrast to our first hypothesis (H1), we found no statistical evidence that temperature was associated with variation in the number or proportion of core and peripheral species (LME, $P > 0.05$; Fig. 1a–c, S3a–c)."

35. Figure 1 E. This model predicts a negative flux at 5C, which cannot be true, thus this model does not seem like the best one to describe these data. Why not fit an exponential model (following theory) via logging temperature?

R35: We believe you are referring to Fig. 1F. We appreciate this suggestion, which was also raised by Reviewer #2 (please see the Response 4). In response, we explored alternative model specifications, including nonlinear forms. A quadratic model provided a substantially better fit than the linear model (ΔAIC > 13), supporting a nonlinear relationship. Based on this comparison, we have updated Fig. 1F to present the fitted relationship from the polynomial model, which better captures the observed pattern. We have clarified this in the Results/Discussion (L120-123) and Methods (L520-524 and L553-561).

36. Fig 2 b and c Fig 3 a,c. Give the regression parameters and uncertainty here. If the goal is simply to show a linear relationship between x and y and not have a fitted model, then just use correlation.. What is the shade on the plots and how estimated? I am guessing the confidence interval of the model.

R36: We now report regression parameters and associated uncertainty in the figure legends (in the main text and supplemental material). We also clarify that shaded areas represent 95% confidence intervals derived from model predictions.

37. 195. Warm streams have lower...

R37: Revised to observational phrasing (L207-210).

Revised text:

"Despite the stabilising role of core inertia, it is important to note that robustness still declined with increasing temperature in all deletion scenarios (Fig. 3c), indicating that warmer streams exhibit lower food web stability regardless of which species are lost."

38. 211 Despite no evidence for an interaction, I suggest that model with terms (0,1) for periphery and random would fit these data lot better than a single model, thus I suggest plotting 3 lines (unless I am wrong, easy to test).

R38: We tested models including interaction terms and scenario-specific slopes. As the interaction between temperature and deletion scenario was not supported statistically, a common slope model was retained for parsimony and interpretability. Scenario-specific models did not improve fit (ΔAIC = 23.64). Thus, we retained a single regression line to emphasise the consistent temperature–robustness relationship across scenarios.

Revised legend:

"Figure 3. Effects of temperature on food web connectance and robustness. (A) Food web connectance declines with increasing stream temperature. (B) Average robustness (R_{50}) of the 14 food webs under three deletion scenarios: core, periphery, and random. Robustness was significantly lower following core species deletions compared to periphery or random deletions (LME: $F_{2,26}$ = 25.69, P < 0.001; p < 0.05, Tukey HSD). (C) Robustness decreases with increasing temperature across all deletion scenarios (LME: $F_{1,12}$ = 14.74, P = 0.002). As no significant interaction was found between temperature and deletion scenario, and an additive model provided a better fit (ΔAIC = 21.64), a single regression line is shown. Shading indicates the 95% confidence interval."

39. 258. Profound over a giant range of temperature

R39: Revised for precision (L318-320).

Revised text:

"Our study reveals how warming drives internal reorganisation of food-web substructures across a broad temperature gradient, particularly within core species that underpin ecosystem functioning and stability."

40. 261. Delete "significant" because it can be confused with p<0.05

R40: Done

41. 325 I thought filamentous algae could be dominant in some of these streams?

R41: We acknowledge that filamentous algae can be locally abundant. However, diatoms were used as the primary basal resource proxy because: (1) they are consistently present across all streams; (2) they dominate basal resource pathways supporting invertebrate consumers, as revealed by our gut content analyses; and (3) they allow standardised, quantitative identification and biomass estimation

Filamentous algae were included in gut content analyses and all food webs but represent a relatively minor flux through the food web.

42. 337 How were prey identified? It is difficult, but certainly possible, to ID prey from parts seen under a microscope

R42: We have clarified the methodology and its reliability (L402-405).

Revised text:

“Prey items were identified from diagnostic morphological remains (e.g. head capsules, mandibles, setae). While partial digestion can limit taxonomic resolution, this approach reliably distinguishes major prey groups and is widely used in aquatic food web studies.”

43. 365. 6 years is not very recent.

R43: Revised wording (L433).

Revised text:

“We applied a robust and widely used approach for estimating energy fluxes through trophic links…”

44. 377, 378. Give units for these. I like to keep rates and fluxes separate for these types of analyses where rate is 1/time and flux is mass/time

R44: We have clarified units as suggested (L446-448).

Revised text:

“Z_i represents the population-level energy loss rate due to metabolic activities and mortality [$J\,h^{-1}$]. F_{ij} denotes the maximum population-level feeding rate of species *j* feeding on species *i* [$J\,h^{-1}$].”

45. 398. One way to test these fluxes is to compare the P/B where P is secondary production and B is biomass for these taxa with the empirically measured P/B in the same streams. I provided citations for these data above.

R45: While direct validation using empirical P/B ratios would be informative, such data were not consistently available for all taxa. The fluxweb approach provides a mechanistically grounded and widely validated framework that integrates metabolic theory and trophic structure.

46. 405. The first term in this model should be equivalent to NPP, for which a coarse estimate is 0.5*GPP. Other studies have measured GPP in these same streams. Are they close?

R46: We appreciate this insightful suggestion. Our framework estimates fluxes at the level of trophic interactions rather than ecosystem metabolism. While producer growth is conceptually linked to NPP, direct comparison with GPP-derived estimates is not straightforward due to differences in scale and measurement in our dataset.

Response Letter – Antiqueira et al. Core reorganisation in food webs buffers ecosystems against warming - Communications Earth & Environment – R2

<u>Author's Note</u>: Below, we provide detailed replies to each point raised by the reviewers. Our responses are presented in boldface, and we indicate the line numbers corresponding to the clean version of the revised manuscript where the changes were made. All modifications within the clean version are highlighted in boldface to enhance clarity and traceability.

REVIEWERS' COMMENTS:

Reviewer #1:
Remarks to the Author:
Comment 1: This is a well considered response to the review and addresses the concerns raised.
R1: Thank you for your positive evaluation of our revised manuscript and for acknowledging that the previous concerns have been satisfactorily addressed.

Reviewer #2:
Remarks to the Author:
Comment 2: The revised manuscript is much improved over the original submission. The authors addressed my biggest concern from the first iteration. They also made several edits that clarify or add context to the previous version. Additionally, Reviewer 3 made several excellent points in their review which were very insightful and made substantial improvements to the manuscript. From my view, the authors attended to the comments from Reviewer 3, although it is possible that I misinterpreted some details. Throughout this document, my new comments will be preceded by "**"

Note, we have shortened the response to the only outstanding comment that may need addressing.

** I appreciate the follow-through related to my concerns about the model fit as plotted in Figure 1F. The updated model reflects the non-linear relationship between temperature and biological processes. I would expect that the function would be exponential, with the flux=0 at temp=0, instead of a second order polynomial based on Q10, but there is evidence that Q10 does not follow a strict exponential relationship (see Mundim et al. 2020, https://doi.org/10.1016/j.ecolmodel.2020.109127, for more information. That said, the polynomial relationship you modelled is likely indistinguishable from an exponential relationship at the lower values, given the error represented in your plot. This model represents a much better fit than the original, linear relationship you modelled so it addresses my concerns.

Response to Reviewer #2

R2: Thank you for the careful re-evaluation of our manuscript and for the positive and constructive feedback. We are pleased that the revisions have improved the clarity and robustness of the work, and we appreciate the thoughtful additional comments.

We appreciate the follow-up regarding the functional form of the temperature–flux relationship (Fig. 1F). We agree that an exponential relationship is well motivated from theory, particularly under the Metabolic Theory of Ecology.

In response to comments from you and Referee #3, we have now explicitly fitted an exponential model by log-transforming energy flux. As expected, this model captures the monotonic increase in flux with temperature and avoids biologically unrealistic predictions. However, it provides a poorer statistical fit compared to the quadratic model (ΔAIC = 42.58) or the linear model (ΔAIC = 29). To balance theoretical expectations with empirical fit, we have revised our presentation as follows: the main text (Fig. 1F) keeps the quadratic (data-driven) model, while the exponential (theory-driven) and linear models is presented as Supplementary Information.

We have clarified this point in the response to Referee #3 below (Response 3), and in the revised manuscript (Results/Discussion L123-129 and Methods L532-542).

Thank you again for the insightful comments, which have helped improve both the clarity and interpretation of the manuscript.

Reviewer #3:
Remarks to the Author:
Comment 3) This manuscript is a revision of one that I reviewed before. The authors addressed my most substantial criticisms. I also checked out the code and data and indeed it was easy to download and peruse, thanks for that.

I also read through my review where I found an awful mistake in my advice:

"Figure 1 E. This model predicts a negative flux at 5C, which cannot be true, thus this model does not seem like the best one to describe these data. Why not fit an exponential model (following theory) via logging temperature?"

I meant logging energy flux (i.e. exponential model)! Logging temperature is a terrible idea, apologies for any confusion. But I would still fit an exponential model as that follows better from theory. Brown et al. 2004, Ecology. Even if it does have \Delta AIC higher than that from a quadratic. I view the goal of this paper is is to test theory, not

compare statistical models. Deviations from theory (via a less than perfect fit) are where we learn.

R3: Thank you for this clarification and helpful suggestion. We agree that an exponential relationship between temperature and energy flux is strongly motivated by theory, particularly under the Metabolic Theory of Ecology. At the same time, a growing body of work indicates that temperature dependencies in ecosystem processes do not always follow a strictly exponential form across environmental gradients, as also noted by Reviewer #2 (e.g. Mundim et al. 2020; Meng et al. 2024; Niu et al. 2024).

In response, we have now explicitly fitted an exponential model by log-transforming energy flux, as recommended. This formulation captures the expected monotonic increase with temperature and avoids biologically unrealistic predictions. However, it provides a poorer statistical fit compared to the quadratic model (ΔAIC = 42.58) or the linear model (ΔAIC = 29).

To balance theoretical expectations with empirical fit, we have revised our presentation as follows: the main text (Fig. 1F) keeps the quadratic (data-driven) model, while the exponential (theory-driven) and linear models are presented in Supplementary Information. This allows direct assessment of how closely the observed relationship aligns with theoretical predictions. Deviations from a strictly exponential form are consistent with evidence that temperature sensitivities (e.g. Q10 temperature coefficient) vary across environmental contexts rather than remaining constant.

We have revised the Results/Discussion (L123-129), and Methods (L532-542) to reflect this comparison and its implications.

L123-129: "Nevertheless, there was an increase in total energy flux with warming (Fig. 1d-f), indicating an overall increase in the strength of trophic interactions in the warmer environment that could potentially bring destabilising effects[38,39]. This increase was nonlinear (LME, quadratic term: $F_{2,11}$ = 55.36, $P < 0.001$), with relatively weak responses at lower temperatures (5–15 °C) and a marked rise at higher temperatures (Fig. 1f), consistent with the temperature dependence of metabolic and interaction rates. We also compared a theory-driven exponential model, following the Metabolic Theory of Ecology[20], with a quadratic formulation. The exponential model captured the expected monotonic increase in flux with temperature (Fig. S4) but provided a poorer fit than the quadratic model (ΔAIC = 22.44). Deviations from strict exponential scaling are consistent with growing evidence that temperature sensitivities (e.g. Q10 temperature coefficient) can vary across environmental gradients rather than remaining constant (e.g. [40–42])."

L532-542: "To evaluate alternative functional forms describing the relationship between temperature and total energy flux, we compared linear, exponential, and quadratic (second-degree polynomial) models. The exponential formulation was implemented by log-transforming energy flux and fitting a linear model against

temperature, consistent with predictions from the Metabolic Theory of Ecology[20]. Model performance was assessed using Akaike's Information Criterion (AIC). The quadratic model provided the best fit ($\Delta AIC_{Polynom-Linear} = 13$, $\Delta AIC_{Polynom-exponential} = 42.28$) and was retained for the main analysis and is presented in the main text (Fig. 1F). The exponential (theory-driven) and linear models are presented in the Supplementary Information as comparative benchmarks (Fig. S4)."

Mundim, K.C., Baraldi, S., Machado, H.G. and Vieira, F.M., 2020. Temperature coefficient (Q10) and its applications in biological systems: Beyond the Arrhenius theory. *Ecological Modelling, 431,* p.109127.

Meng, C., Xiao, X., Wagle, P., Zhang, C., Pan, L., Pan, B., Qin, Y. and Newman, G.S., 2024. Exponential or unimodal relationships between nighttime ecosystem respiration and temperature at the eddy covariance flux tower sites. *Ecology Letters, 27*(10), p.e14532.

Niu, S., Chen, W., Liáng, L.L., Sierra, C.A., Xia, J., Wang, S., Heskel, M., Patel, K.F., Bond-Lamberty, B., Wang, J. and Yvon-Durocher, G., 2024. Temperature responses of ecosystem respiration. *Nature Reviews Earth & Environment, 5*(8), pp.559-571.

Comment 4) This answer needs better justification "While direct validation using empirical P/B ratios would be informative, such data were not consistently available for all taxa. The fluxweb approach provides a mechanistically grounded and widely validated framework that integrates metabolic theory and trophic structure."

I agree with the authors that such data are not available for all taxa. But they exist for common taxa and indeed for the entire assemblage (Table 1 Nelson et al. Ecology). How does productivity and thus P/B derived from the model compare for some of the common taxa or most of the assemblage? I am not looking for a close relationship. But if off by 100 or 1000, then I would want to know why.

Ditto on the NPP. I am not sure what "conceptually linked to NPP" means. The sum producer production should be something less than NPP (given not all photoautotrophs were assessed), and NPP is probably close to 0.5* GPP. Again, the goal is to not for these fluxes to closely match heree, but not off by 1000 either.

R4: We appreciate the opportunity to clarify this point.

We believe there may be a misunderstanding regarding the interpretation of the energy fluxes estimated in our study. The quantities derived using the *fluxweb* framework do not represent secondary production. Rather, they quantify energy fluxes between interacting species, i.e. the rate at which energy (or carbon) is

transferred from resources to consumers. These fluxes are therefore more closely related to interaction strengths within the food web than to population-level production rates.

Consequently, dividing these fluxes by biomass to derive quantities analogous to P/B ratios would not be conceptually appropriate. P/B ratios quantify biomass generation and turnover at the population level (i.e. production per unit biomass), whereas the fluxes we estimate represent trophic transfers across links in the network. These are fundamentally different ecological quantities, and we are therefore uncertain how a direct comparison between P/B ratios and "per unit biomass interaction strengths" could be meaningfully interpreted.

To better anchor our estimates empirically, we note that total energy flux (i.e. the sum of all trophic transfers in the network) can be interpreted as the total amount of carbon flowing through the system per unit time. At the ecosystem level, this quantity is conceptually closer to integrative processes such as ecosystem respiration. Importantly, previous work in the same Hengill stream system has demonstrated that total energy flux estimated using the same framework is strongly correlated with empirically measured ecosystem respiration, derived from whole-stream oxygen measurements under light and dark conditions (Kordas et al. 2022; see Supporting Information). This provides an independent, system-level validation of the flux estimates.

We have now clarified this distinction in the revised manuscript and added text to the Methods (L475-483) and Discussion (L129-136) to explicitly explain (i) the difference between energy flux and production-based metrics, and (ii) the empirical support for flux-based estimates from independent measurements of ecosystem functioning.

We hope that this clarification helps address your concern and provides reassurance regarding the ecological realism and empirical grounding of our approach.

L129-136:

"At the ecosystem level, total energy flux represents the rate at which carbon is processed through the food web, linking these estimates to integrative measures of ecosystem functioning. Notably, previous work in the same Hengill stream system has shown that flux-based estimates are strongly correlated with independently derived measures of ecosystem respiration from whole-stream oxygen dynamics[9], providing empirical support for their ecological realism and relevance as system-level indicators of energy processing."

L475-483:

“Temperature-dependent parameterisations follow established metabolic theory and are implemented within the widely used fluxweb framework[69], with prior validation in the same stream food web system[9]. Importantly, these estimates represent the rate of energy transfer between resources and consumers (i.e. interaction strengths), rather than population-level production. As such, they are not directly comparable to metrics such as secondary production or P/B ratios, which describe biomass generation and turnover within taxa. Comparable production-based estimates have been derived independently in the same study system (e.g. [71]).”

Kordas, R.L., Pawar, S., Kontopoulos, D.G., Woodward, G. and O’Gorman, E.J., 2022. Metabolic plasticity can amplify ecosystem responses to global warming. *Nature Communications, 13*(1), p.2161.
