# Supplementary material for: Core reorganisation in food webs buffers ecosystems against warming
Source: Commun Earth Environ. 2026 Jul 16;7(1):874. doi: 10.1038/s43247-026-03813-4 (PMC13745283; doi:10.1038/s43247-026-03813-4)
Supplement: Supplementary file 2 [file 43247_2026_3813_MOESM2_ESM.pdf]

Supplemental Material

Core reorganisation in food webs buffers ecosystems against warming

Pablo A. Poleto Antiqueira[1], Athen Ma[2], Pavel Kratina[3], Vito Latora[2], Lachlan G. Burton[2], Lei Zhao[4], Eoin J. O'Gorman[1]

1 School of Life Sciences, University of Essex, Wivenhoe Park, Colchester, CO4 3SQ, UK

2 Queen Mary University of London, Mile End Road, London, E1 4NS, UK

[3] Centre for Biodiversity and Sustainability, School of Biological and Behavioural Sciences, Queen Mary University of London, Mile End Road, London E1 4NS, UK

[4] Beijing Key Laboratory of Biodiversity and Organic Farming, College of Resources and Environmental Sciences, China Agricultural University, Beijing, China

Supplementary Table 1. List of species and their trophic category. Their distributions in the core and periphery in Hengill geothermal streams food webs are indicated by the Stream ID.

Species/biological group	Trophic category	Stream ID	
		Core	Periphery
Achnanthes	Resource	1,3,4,5,6,7,9,10,11,12,13,14,16	8
Amphora	Resource	1,4,5,10	3,6,7,8,9,11,12,13,14,16
Asterionella	Resource		9
Aulacoseira	Resource		5,6
Caloneis	Resource		3,4,5,12,14
Capnia vidua	Consumer/ Resource	10,12,13,16	
Chaetocladius sp.	Consumer	3,4,6,9	
Clinocera stagnalis	Consumer/ Resource		4,7,10,11,13,14,16
Cocconeis	Resource	1,3,4,5,6,7,9,10,11,13,14,16	8
CPOM	Resource	4,5,10,11,13,16	1,3,6,7,8,9,12,14
Cricotopus sp. A	Consumer	5,8,13	
Cricotopus sp. B	Consumer	3,7,11,14	
Cymbella	Resource	3,4,5,10	6,7,8,9,11,12,13,14,16
Cyclotella	Resource		5,8,9
Diamesa bertrami	Consumer	7,10,13,14,16	
Diamesa zernyi	Consumer	7,10,13,14,16	
Diatoma	Resource	3,4,5,6,7,8,9,10,11,12,13,14,16	
Dicranota exclusa	Consumer	7,10,11,13	
Diploneis	Resource		4,5,6,11,12,16
Epithemia	Resource	3,4,5,6,9,11,12,14,16	
Eukiefferiella claripennis	Consumer	3	
Eukiefferiella minor	Consumer	1,4,5,6,7,8,9,10,11,12,13,14,16	
Eunotia	Resource		1,9,11,13
Filamentous algae	Resource		1,3,4,5,6,7,8,9,10,11,12,13,14,16
FPOM	Resource	1,3,4,5,6,7,9,10,11,12,13,14,16	8
Fragilaria	Resource	1,4,5,6,7,9,10,11,13,14,16	3,8,12
Frustulia	Resource	4	1,12,13,14
Gomphonema	Resource	1,3,4,5,6,7,9,10,11,12,14,16	8
Green algae	Resource		1,3,4,5,6,7,8,9,10,11,12,13,14,16
Hebridae	Consumer		8
Helobdella stagnalis	Consumer/ Resource		1
Hemiptera	Consumer		3
Hydrellia sp.	Consumer	5	
Limnophora riparia	Consumer/ Resource	1,3,4,5,6,8,9,14,16	11,12
Limoniinae sp.	Consumer	6,10	
Macropelopia sp.	Consumer	3,12	
Macrophytes	Resource	9,11	1,3,6,7,8,12,13,14
Melosira	Resource	3,4,5,9,10,11,12	1,6,8,14

Meridion	Resource	1,3,4,5,6,7,9,10,11,12,13,14,16	8
Mesoveliidae spA	Consumer		5,7,11,13
Mesoveliidae spB	Consumer	5	5
Metriocnemus sp. A	Consumer	7,9,13	
Metriocnemus sp. B	Consumer	3,4,7,16	
Micropsectra atrofasciata	Consumer	1,3,4,5,6,7,8,9,10,11,13,14,16	
Naucoridae	Consumer		8
Navicula	Resource	1,3,4,5,10	6,7,8,9,11,12,13,14,16
Nitzschia	Resource	1,4,5,10	3,6,7,8,9,11,12,13,14,16
Notonectidae	Consumer		11
Oligochaeta sp	Consumer/ Resource	1,3,4,5,6,7,8,9,10,11,12,13,14,16	
Opephora	Resource		5,10,16
Orthocladius sp.	Consumer/ Resource	1,5,8,12,13,14	
Paracladopelma sp.	Consumer/ Resource	3	
Paracricotopus sp.	Consumer/ Resource	10	
Pinnularia	Resource	3	1,4,6,9,10,11,12,13,14,16
Pisidium sp.	Consumer/ Resource	1	
Potamophylax cingulatus	Consumer/ Resource	7,9,11,12,13,14,16	
Prionocera turcica	Consumer	12,13	
Procladius islandicus	Consumer/ Resource	4,6,9	
Prosimulium ursinum	Consumer/ Resource	7	
Radix balthica	Consumer/ Resource	1,3,4,5,6,7,8,9,11,12,16	
Rheocricotopus effesus	Consumer/ Resource	4,7,9,10,11,12,13,14,16	
Rhoicosphenia	Resource	1,3,4,5,6	7,8,9,11,12,14,16
Rhopalodia	Resource	1,3,4,5,6,9,11,12,14,16	7,8
Salmo trutta	Consumer	1,3,5,6,12,16	8
Scatella sp.	Consumer		4,7,11
Simulium aureum	Consumer/ Resource	1,3,4,5,6,7,8,9,10,11,12,14,16	
Simulium vittatum	Consumer/ Resource	1,3,4,5,6,7,8,9,10,11,14,16	
Sperchon glandulosus	Consumer/ Resource		1,3,4,5,6,7,9,10,11,12,13,14,16
Stauroneis	Resource		5,7
Surirella	Resource		4,12,16
Synedra	Resource	4,5,6,7,8,9,11,12,13,14,16	
Terrestrial resources	Resource		1,3,4,5,6,7,8,9,10,11,12,13,14,16
Tetracyclus	Resource		1
Thienemanniella sp.	Consumer/ Resource	1,4,5,6,7,9,10,11,12,13,14,16	
Turbellaria	Consumer		5

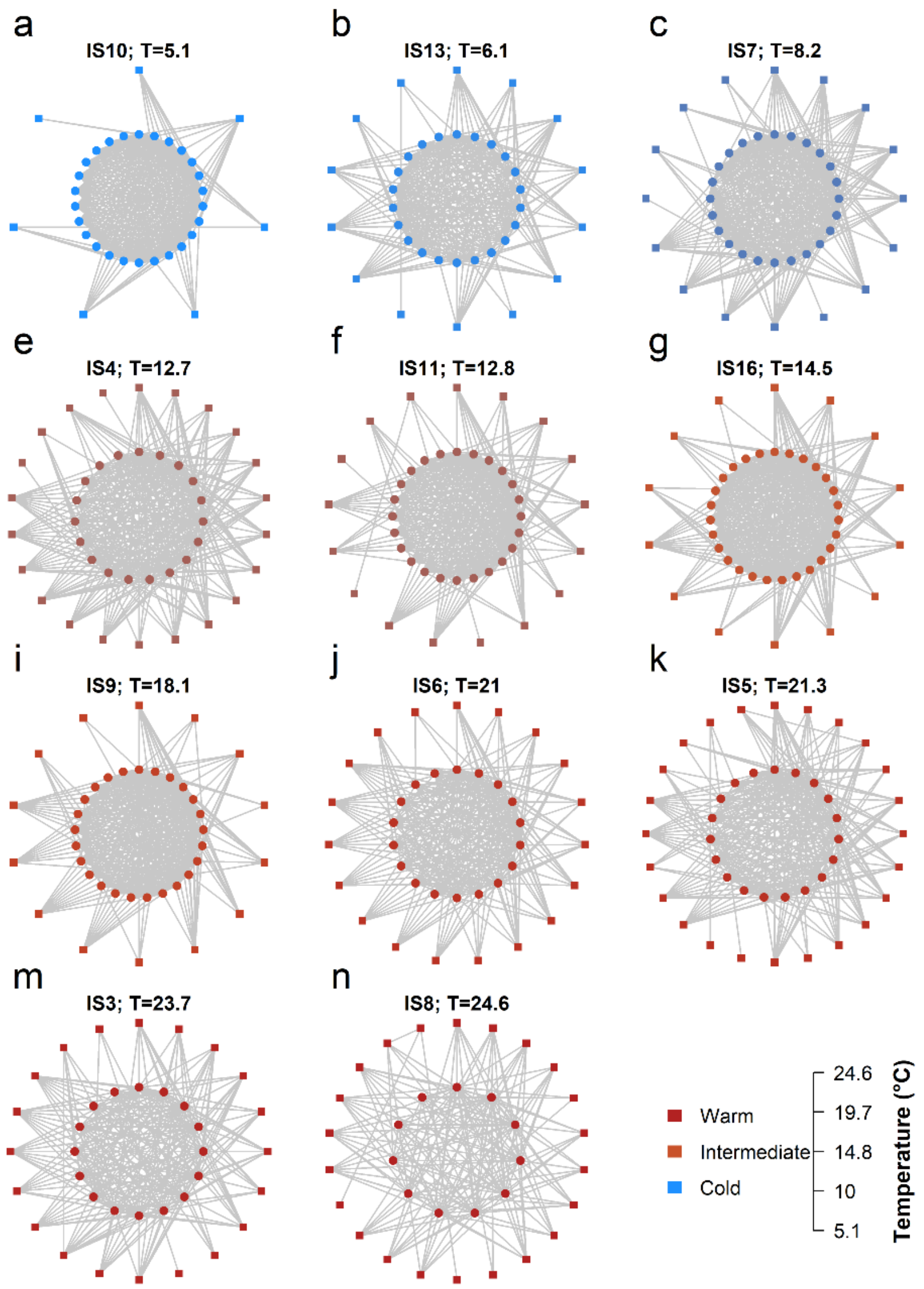

Supplementary Figure 1. Core-Periphery structure of ecological networks sorted by temperature. Core-periphery structure of multiple ecological networks from 14 study streams, ordered by increasing temperature. Each network is plotted in a circular layout with nodes positioned according to their connectivity. Core nodes (highly connected species) are represented as circles in the inner ring are surrounded by peripheral species (less connected species) as squares in the outer ring.

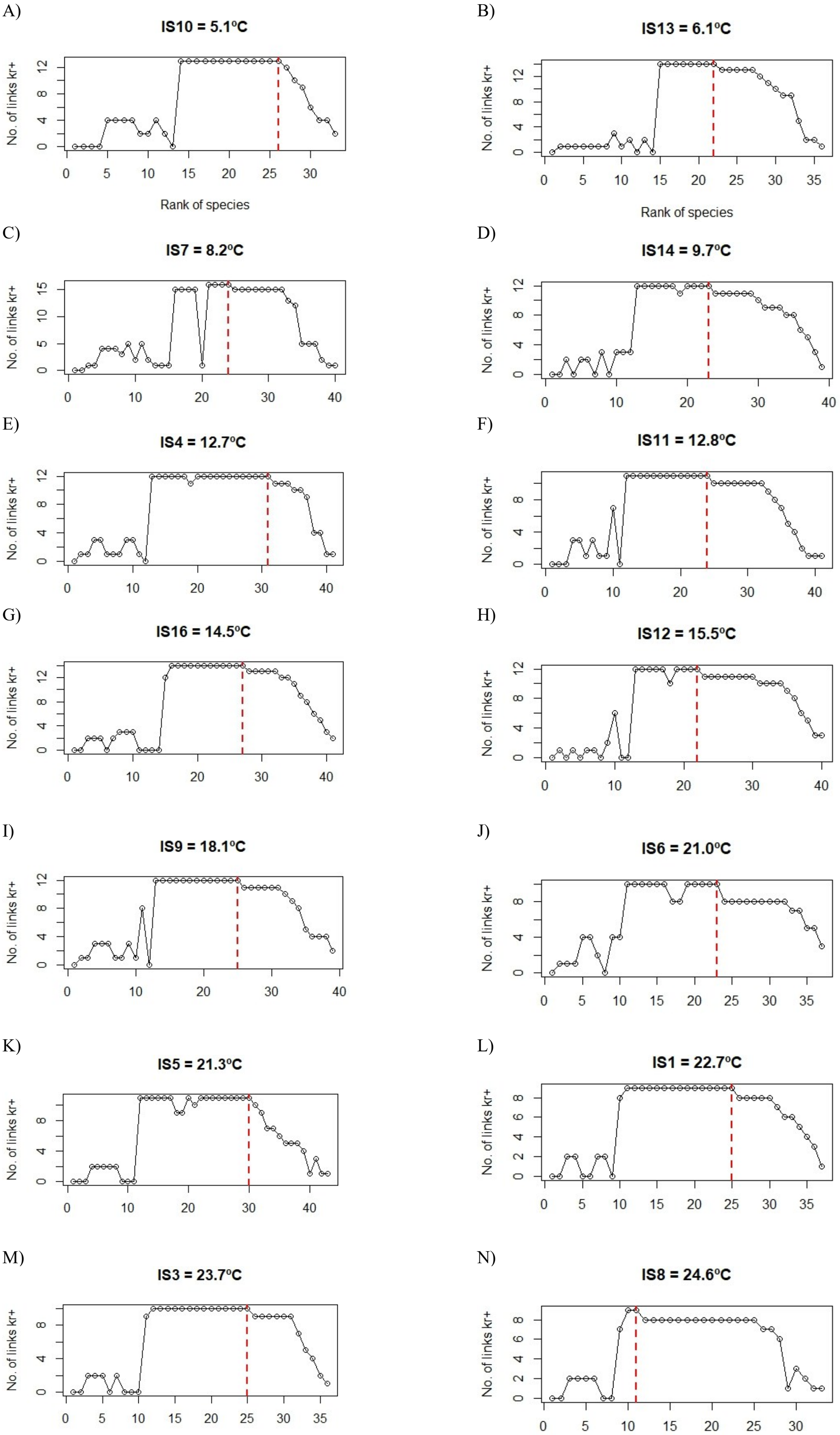

Supplementary Figure 2. Comparisons of core profiles from 14 geothermal streams food webs in an increasing order of temperature (a-n). Nodes are ranked by their decreasing order of degree and plotted by the number of links with nodes of a higher rank, k^{+}_{r}. The maximum of the curve k^{+}_{r*} defines the boundary of the core, which is indicated by a vertical red line.

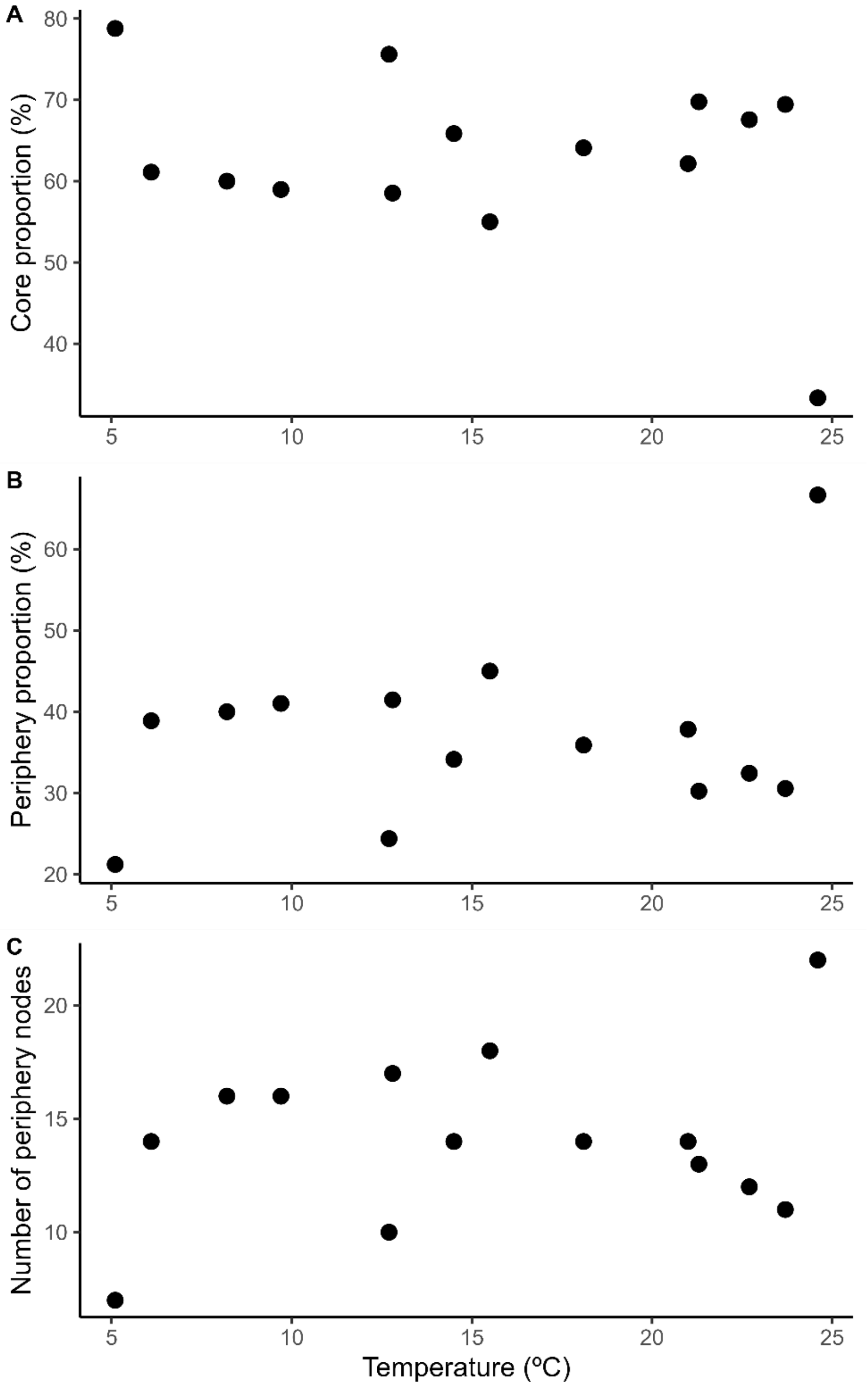

Supplementary Figure 3. Temperature effects on core–periphery structure in stream food webs. (A) Proportion of core species, (B) proportion of peripheral species, and (C) absolute number of peripheral nodes plotted against stream temperature (°C). Each point represents a food web from a distinct stream site.

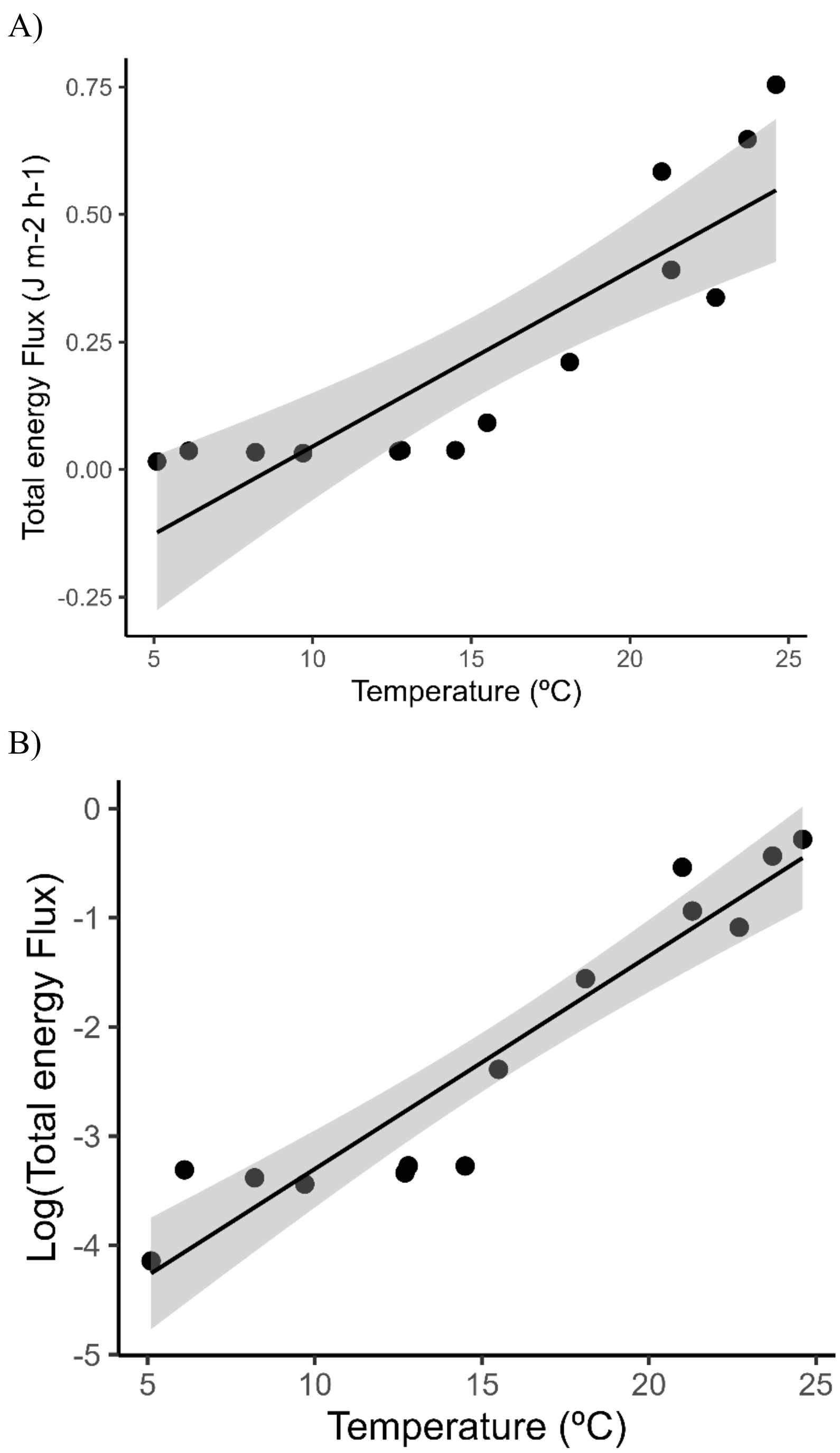

Supplementary Figure 4. Temperature dependence of total energy flux across stream food webs. (A) Relationship between temperature and total energy flux estimated using a linear mixed-effects model. (B) The same relationship modelled using an exponential formulation (linear model fitted to log-transformed energy flux). Points represent individual stream sites. Solid lines show fitted relationships (Linear model: y= -0.29 + 0.03x, Exponential model: y= -5.25 + 0.19x), and shaded areas indicate 95% confidence intervals. Temperature had a significant positive effect on total energy flux in both formulations (linear model: $F_{1,12}$ = 35.79, P < 0.0001, exponential model: $F_{1,12}$ = 102.08, P < 0.0001).

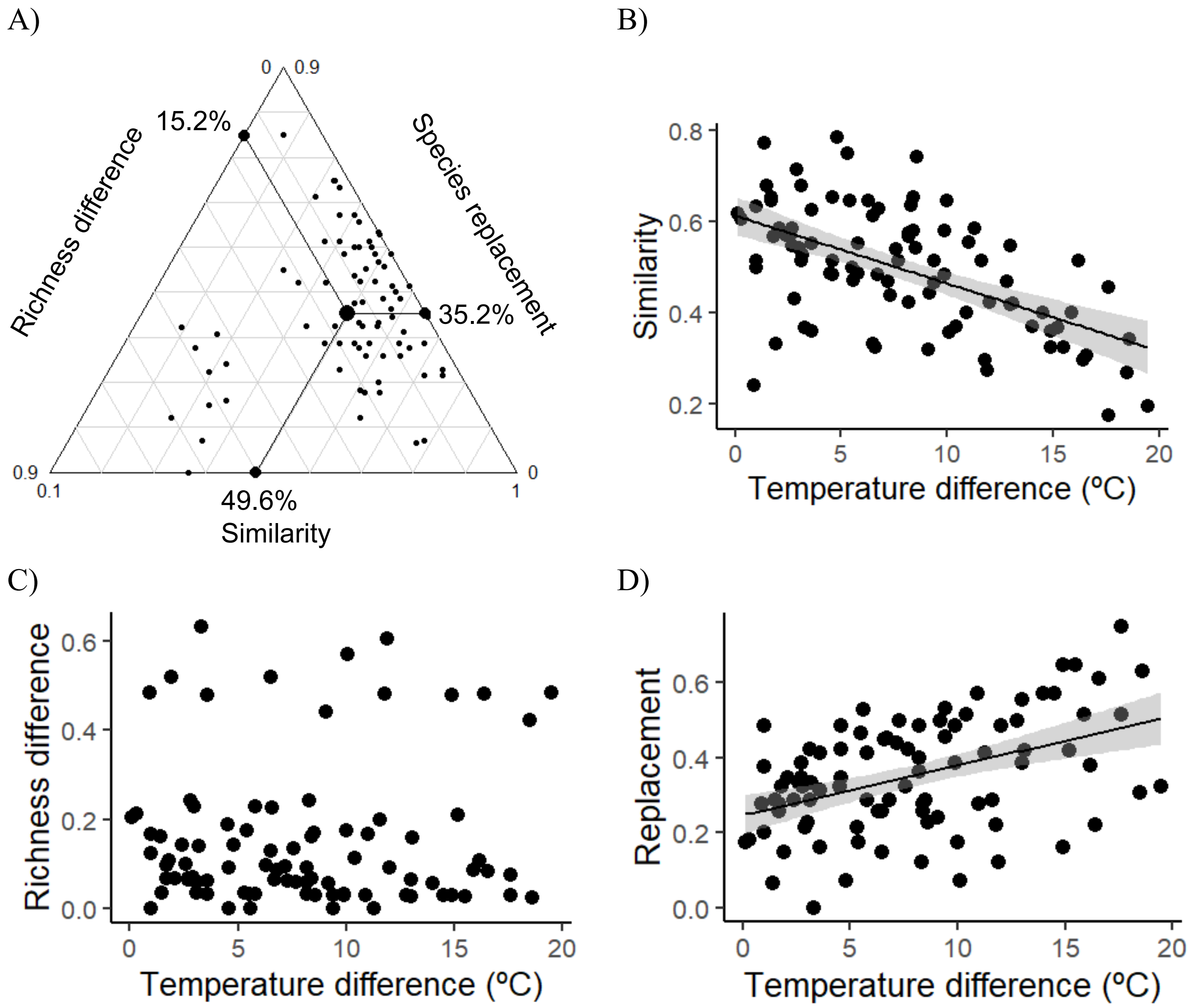

Supplementary Figure 5. Temperature-driven variation in presence–absence-based beta diversity of core food web nodes. a) SDR-simplex plots from pairwise comparisons across the 14 streams, showing the relative contributions of species similarity (1 – total β diversity), species replacement, and species richness difference among core nodes. Each point represents a pair of sites. The large central dot in the triangle represents the centroid of the points. The values are based on the Jaccard dissimilarity matrix for species occurrence data (presence/absence). B-D) Beta diversity across the temperature gradient: b) total beta diversity, c) richness difference component of beta diversity and d) species replacement component of beta diversity. The solid line represents the fitted relationship from linear models (LM), and the shaded area indicates the 95% confidence interval. Temperature had a significant effect on similarity ($y = 0.613 - 0.015x$; LM: $F_{1,89} = 41.67$, $R^2 = 0.31$, $P < 0.001$) and species replacement ($y = 0.247 + 0.013x$; LM: $F_{1,89} = 22.47$, $R^2 = 0.19$, $P < 0.001$).

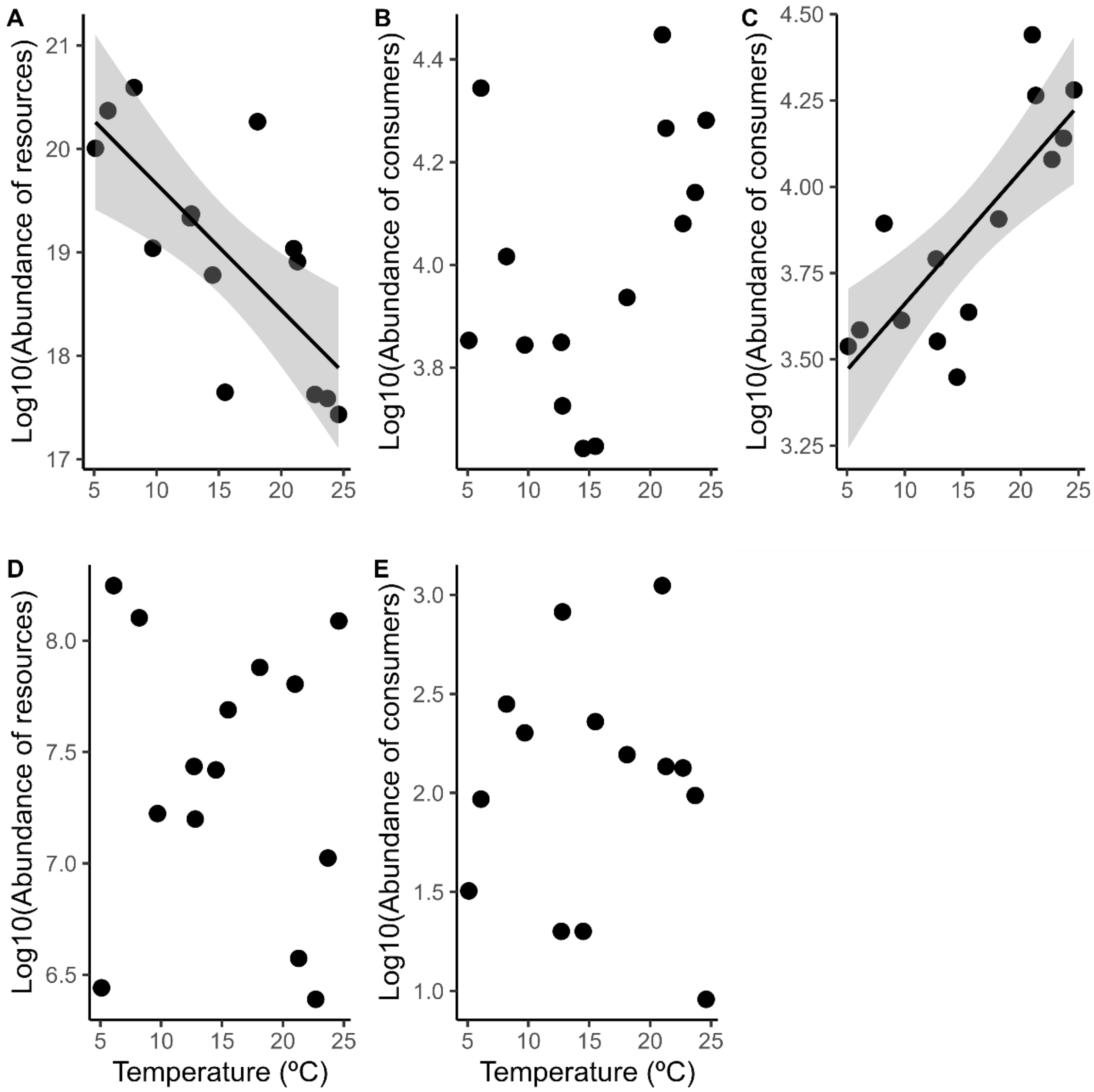

Supplementary Figure 6. Changes in the abundance of consumers and resources in the core (top) and periphery (bottom). (a) There was a significant decrease in the abundance of core resources with increasing temperature ($y = 9.07 - 0.05x$; Linear Model, P=0.002, R^2=0.51). (b) There was no change in the abundance of core consumers with increasing temperature (Linear Model, P=0.173, R^2=0.08). The chironomid *Eukiefferiella minor* was super-abundant in the coldest stream (possibly due to lack of competition from larger diatom grazers such as the snail *Radix balthica*), however, and when this species was removed, there was (c) a significant increase in core consumers with increasing temperature ($y = 3.277 + 0.038x$; Linear Model, P<0.001, R^2=0.58). (d) There was no change in the abundance of periphery resources with increasing temperature (Linear Model, P=0.651, R^2= -0.06). (e) There was no change in the abundance of periphery consumers with increasing temperature (Linear Model, P=0.947, R^2= -0.08).

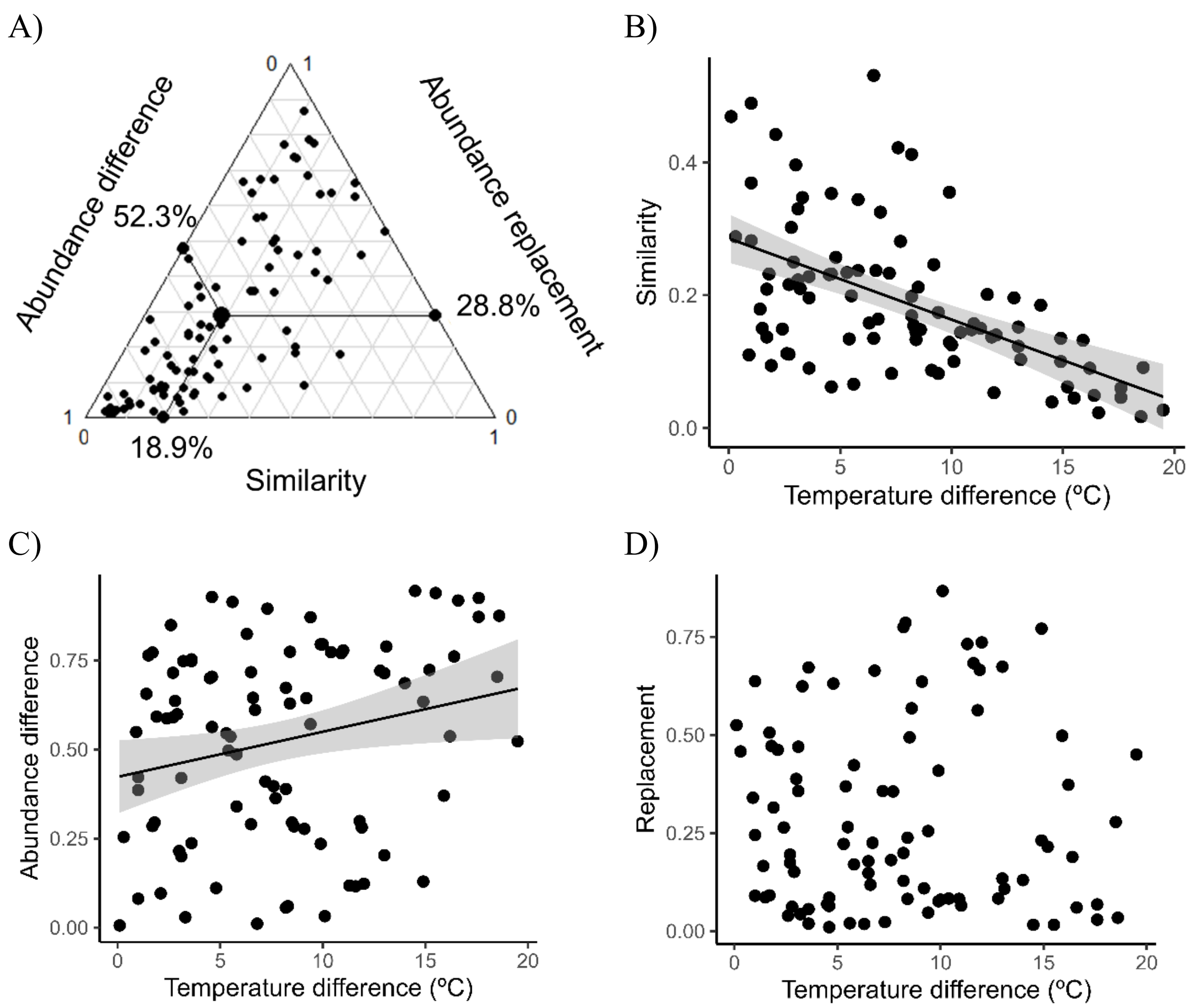

Supplementary Figure 7. Temperature-driven variation in abundance-based beta diversity of entire food webs. a) SDR-simplex plots based on comparisons of whole food web nodes (not just core) across 14 geothermal streams, partitioned into species similarity (1 – β diversity), abundance difference, and abundance replacement. Values use the Ruzicka dissimilarity matrix for abundance data. Each point represents a pair of sites. The large central dot in the triangle represents the centroid of the points. The values are based on the Ruzicka dissimilarity matrix for abundance data. B-D) Beta diversity across the temperature gradient: b) total similarity (total β diversity – 1), c) abundance difference component of beta diversity and d) abundance replacement component of beta diversity. The solid line represents the fitted relationship from linear models, and the shaded area indicates the 95% confidence interval. Temperature had a significant effect on similarity ($y = 0.285 - 0.012x$; LM: $F_{1,89} = 38.92$, R^2=0.30, $P < 0.001$) and abundance difference ($y = 0.423 + 0.013x$; LM: $F_{1,89} = 5.30$, R^2=0.04, $P = 0.024$).

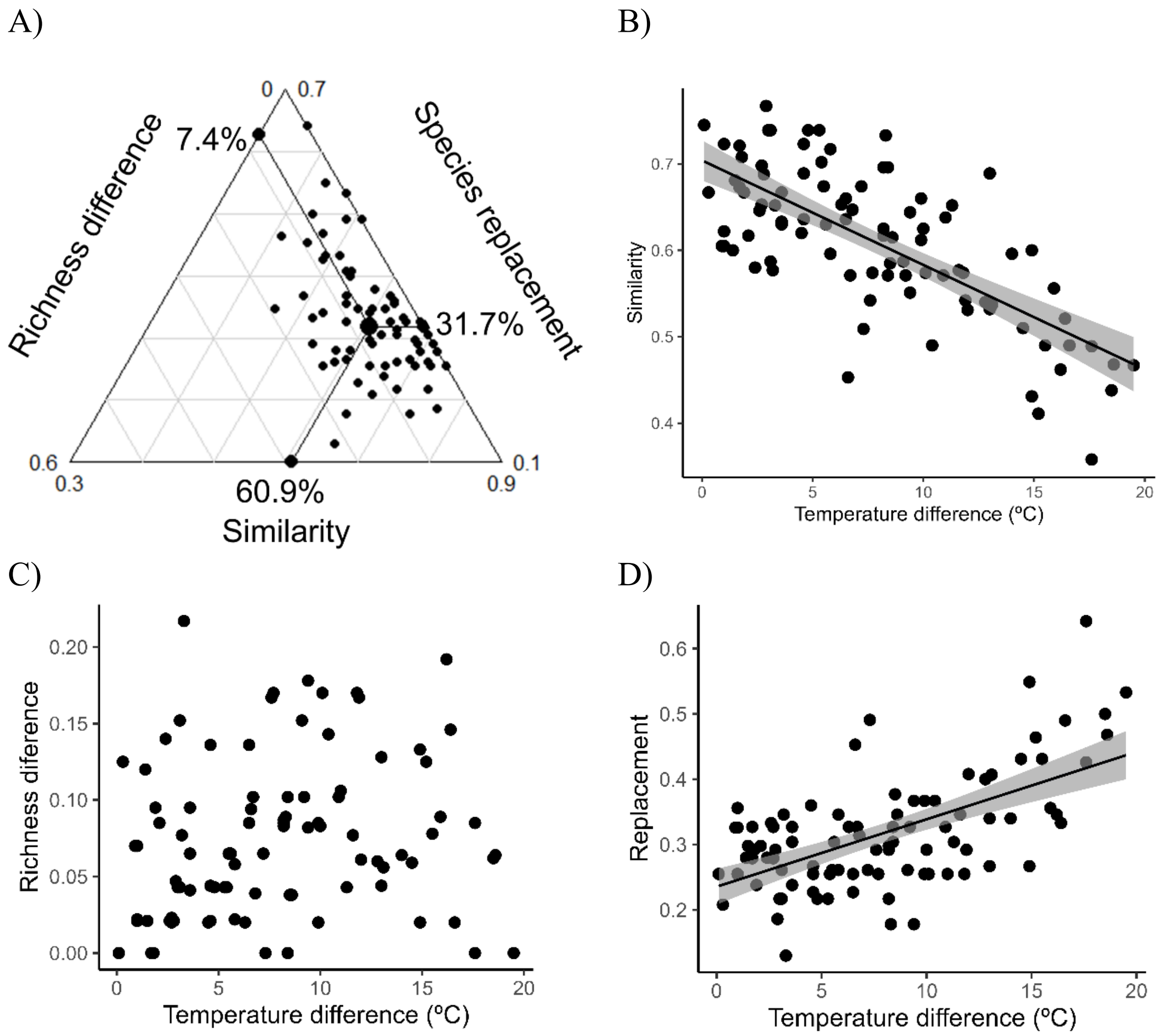

Supplementary Figure 8. Temperature-driven variation in presence–absence-based beta diversity of entire food webs. a) SDR-simplex plots based on comparisons of whole food web nodes (not just core) across 14 geothermal streams, partitioned into species similarity (1 – β diversity), species replacement, and species richness difference. Each point represents a pair of sites. The large central dot in the triangle represents the centroid of the points. The values are based on the Jaccard dissimilarity matrix for species occurrence data (presence/absence). B-D) Beta diversity across the temperature gradient: b) total beta diversity, c) richness difference component of beta diversity and d) species replacement component of beta diversity. The solid line represents the fitted relationship from linear models, and the shaded area indicates the 95% confidence interval. Temperature had a significant effect on similarity ($y = 0.704 - 0.012x$; LM: $F_{1,89} = 93.95$, $R^2=0.51$, $P < 0.001$) and species replacement ($y = 0.235 + 0.010x$; LM: $F_{1,89} = 49.77$, $R^2=0.35$, $P < 0.001$).

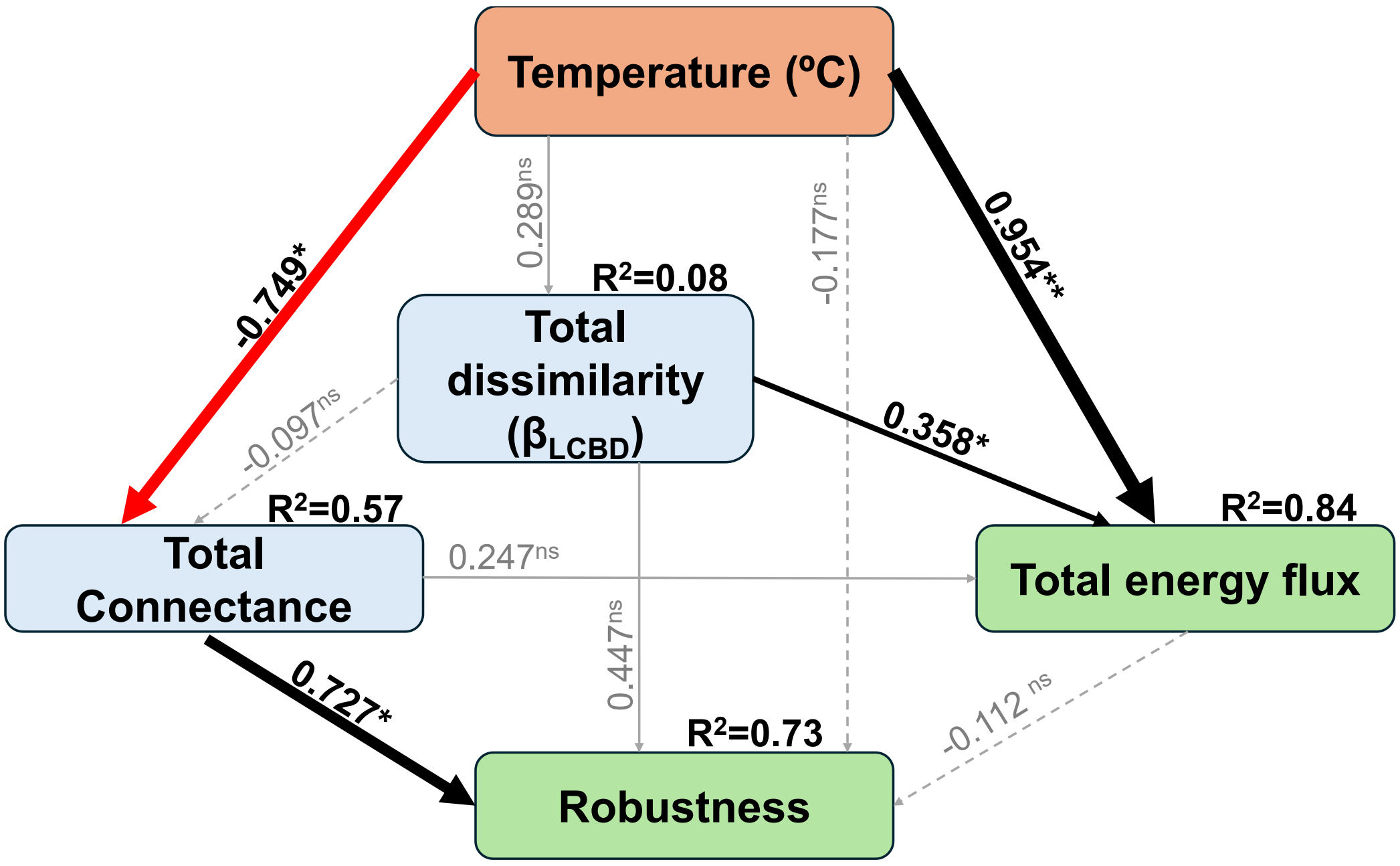

Supplementary Figure 9. Structural equation model linking temperature, node dissimilarity, total connectance, total energy flux and robustness in stream food webs. Standardised path coefficients from piecewise structural equation modelling (pSEM) illustrate the direct and indirect effects of temperature (°C) on food web node dissimilarity (β_{lcbd}), food web connectance, total energy flux and robustness. Black end red arrows denote positive and negative relationships respectively; grey arrows denote non-significant relationships. Arrow thickness corresponds to the magnitude of standardised effects, with asterisks indicating statistical significance (*P < 0.05, **P < 0.01). R^2s for component models are given on the boxes of endogenous variables.

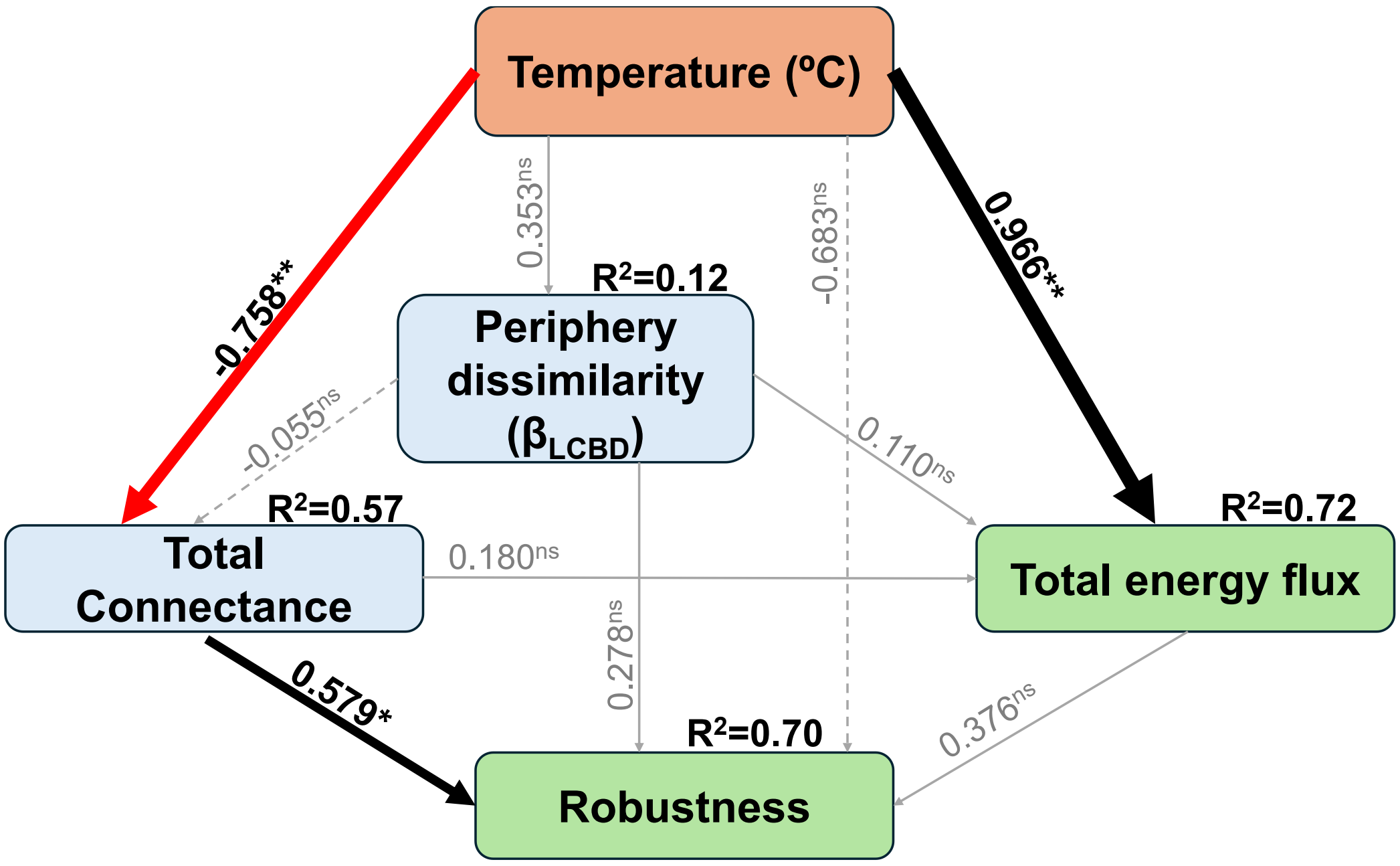

Supplementary Figure 10. Structural equation model linking temperature, periphery node dissimilarity, total connectance, total energy flux and robustness in stream food webs. Standardised path coefficients from piecewise structural equation modelling (pSEM) illustrate the direct and indirect effects of temperature (°C) on periphery node dissimilarity (β_{lcbd}), food web connectance, total energy flux and robustness. Black end red arrows denote positive and negative relationships respectively; grey arrows denote non-significant relationships. Arrow thickness corresponds to the magnitude of standardised effects, with asterisks indicating statistical significance (*P < 0.05, **P < 0.01). R^2s for component models are given on the boxes of endogenous variables.
